# Actinosomes: Condensate-Templated Containers for Engineering Synthetic Cells

**DOI:** 10.1021/acssynbio.2c00290

**Published:** 2022-08-10

**Authors:** Ketan A. Ganar, Liza Leijten, Siddharth Deshpande

**Affiliations:** Laboratory of Physical Chemistry and Soft Matter, Wageningen University and Research, Stippeneng 4, 6708 WE Wageningen, The Netherlands

**Keywords:** Synthetic cells, liquid-liquid phase separation, biomolecular condensates, actin cytoskeleton, cell-free expression

## Abstract

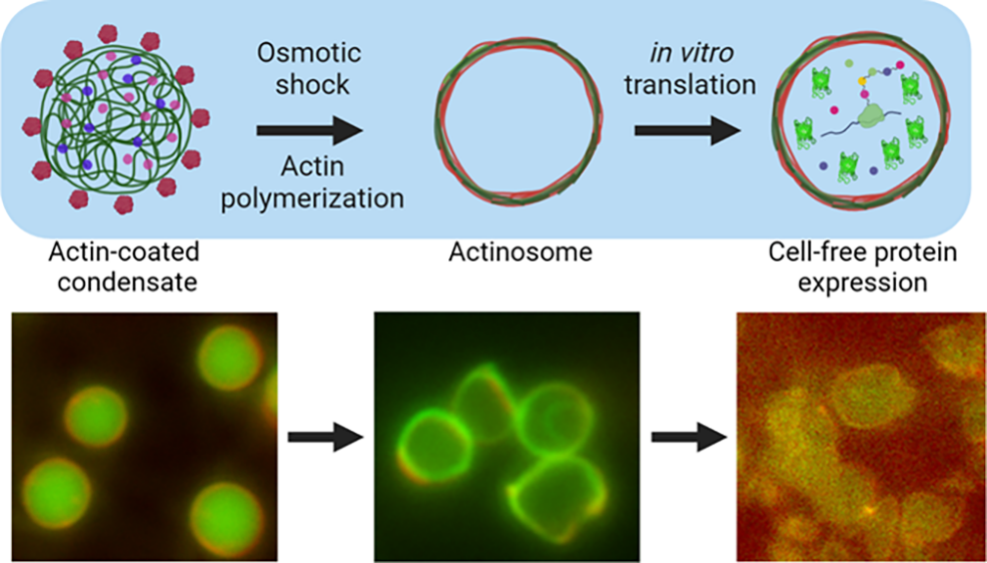

Engineering synthetic cells has a broad appeal, from understanding living cells to designing novel biomaterials for therapeutics, biosensing, and hybrid interfaces. A key prerequisite to creating synthetic cells is a three-dimensional container capable of orchestrating biochemical reactions. In this study, we present an easy and effective technique to make cell-sized porous containers, coined actinosomes, using the interactions between biomolecular condensates and the actin cytoskeleton. This approach uses polypeptide/nucleoside triphosphate condensates and localizes actin monomers on their surface. By triggering actin polymerization and using osmotic gradients, the condensates are transformed into containers, with the boundary made up of actin filaments and polylysine polymers. We show that the guanosine triphosphate (GTP)-to-adenosine triphosphate (ATP) ratio is a crucial parameter for forming actinosomes: insufficient ATP prevents condensate dissolution, while excess ATP leads to undesired crumpling. Permeability studies reveal the porous surface of actinosomes, allowing small molecules to pass through while restricting bigger macromolecules within the interior. We show the functionality of actinosomes as bioreactors by carrying out *in vitro* protein translation within them. Actinosomes are a handy addition to the synthetic cell platform, with appealing properties like ease of production, inherent encapsulation capacity, and a potentially active surface to trigger signaling cascades and form multicellular assemblies, conceivably useful for biotechnological applications.

## Introduction

Cells are highly complex systems consisting of a plethora of interconnected biomolecular networks, and this greatly limits our understanding of how they work. While deciphering molecular mechanisms in living systems is tedious, the *in vitro* reconstitution assay is an excellent complementary approach to studying specific cellular modules. In recent years, the bottom-up construction of synthetic cells has received tremendous attention, where compartmentalization is seen as an essential feature to mimic nature’s way of organizing reactions and, at the same time, providing a superior control.^1^ Synthetic cells typically refer to an enclosed three-dimensional structure capable of performing tasks similar to their biological counterparts. Different types of synthetic cells have been proposed, which can be broadly classified as membrane-bound and membraneless confinements.^2,3^

Membrane-bound compartments, built by the self-assembly of amphiphilic molecules, have been widely used as cell-mimicking prototypes.^4^ This has led to the design of a wide variety of confinements such as surfactant-stabilized water-in-oil droplets, liposomes with a lipid bilayer as the boundary, and even completely synthetic containers such as polymersomes and dendrimersomes.^5,6^ These compartments are capable of reconstituting various biochemical processes within them and have been exploited to engineer a wide variety of cellular modules and to advance various applications like cell-free gene expression,^7,8^ evolving proteins by directed evolution,^9^ cytoskeleton assembly,^10,11^ growth and division,^12−14^ cargos for drug delivery,^15^ and printing artificial tissues.^16,17^ In these confinements formed *via* the hydrophobic effect,^18^ the membrane usually acts as a physical barrier and restricts passive transport of molecules across them. This is commonly resolved by incorporating transmembrane proteins like α-hemolysin, making them selectively permeable.^17,19^ Additionally, newer strategies have been designed such as proteinosomes, which have a membrane comprising cross-linked, amphiphilic protein–polymer conjugates.^20^ Unlike the relatively inert membranes of liposomes and polymersomes, the proteinaceous boundary of proteinosomes can perform enzymatic reactions.^21^ Methods to produce the above-mentioned confinements suffer from various limitations: easy-to-use bulk methods have poor process control, high polydispersity (variation in the confinement size), and a low encapsulation efficiency. Employing microfluidic emulsion-based techniques effectively solve these issues, but at the cost of technologically advanced sophisticated and less-accessible setups.^22,23^

Biomolecular condensates, membraneless structures formed *via* the process of liquid–liquid separation (LLPS), have emerged as new types of synthetic bioreactors in recent years.^24^ After their discovery and realization of the prominent role they play in intracellular biochemistry, they have been heavily exploited also in the realm of synthetic biology. Some salient features of condensates are their ability to sequester molecules and their assemblies,^25^ resistant to extreme conditions,^26^ performing biochemical reactions with increased reaction rates and enhanced enzyme kinetics,^27−29^ and exchange of molecules with their surroundings.^24^ Interestingly, condensates have been explored as possible scaffolds to form synthetic containers.^30^ For example, complex coacervates have been forged into multilayered compartments *via* a surface-templating procedure, albeit producing thick shells and the use of chemical treatments.^31^ Another study demonstrated that the condensates formed by glutamic acid-rich leucine zipper and arginine-rich leucine zipper could be transitioned into hollow vesicles *via* temperature changes.^32^ Alternatively, coacervate droplets can be coated with amphiphilic molecules; small unilamellar lipid vesicles were assembled at the interface of RNA/peptide droplets, transforming them into an RNA-encapsulated membrane-bound confinement.^33^ These studies highlight the potential of condensates as templates to form novel confinements but also present several limitations such as thick shells, low membrane permeability, and use of sophisticated protein engineering. If possible, one would desire a highly biocompatible proteinaceous confinement produced in a straightforward manner, without the use of complicated setups.

In this study, we present a straightforward bottom-up approach to make cell-sized (2–5 μm) confinements with proteins as the building blocks. We start with condensates made up of a polypeptide (polylysine, polyK) and nucleoside triphosphates (NTPs), a mixture of adenosine triphosphate (ATP) and guanosine triphosphate (GTP). We then use actin, the well-known cytoskeletal protein capable of forming filaments, to structurally modify the condensate droplets. Actin localizes at the condensate interface and rapidly polymerizes into filaments at the expense of a high concentration of ATP present in condensates. Under the right conditions, this leads to internal coacervate dissolution, followed by colocalization of polylysine with actin filaments at the surface, resulting in hollow containers, which we term actinosomes. We show that the ATP:GTP ratio is crucial in actinosome assembly, and permeability assays reveal actinosomes as stable, porous containers. Finally, we show the capability of actinosomes as bioreactors by carrying out *in vitro* translation of proteins. We believe the addition of actinosomes, which can be formed without any use of sophisticated setups and in a rapid manner, will be highly useful in the field of synthetic cells and to reconstitute reactions within cell-sized, biocompatible containers.

## Results

### Interaction of Actin with Multicomponent Condensates forms Actinosomes

**Figure 1:**
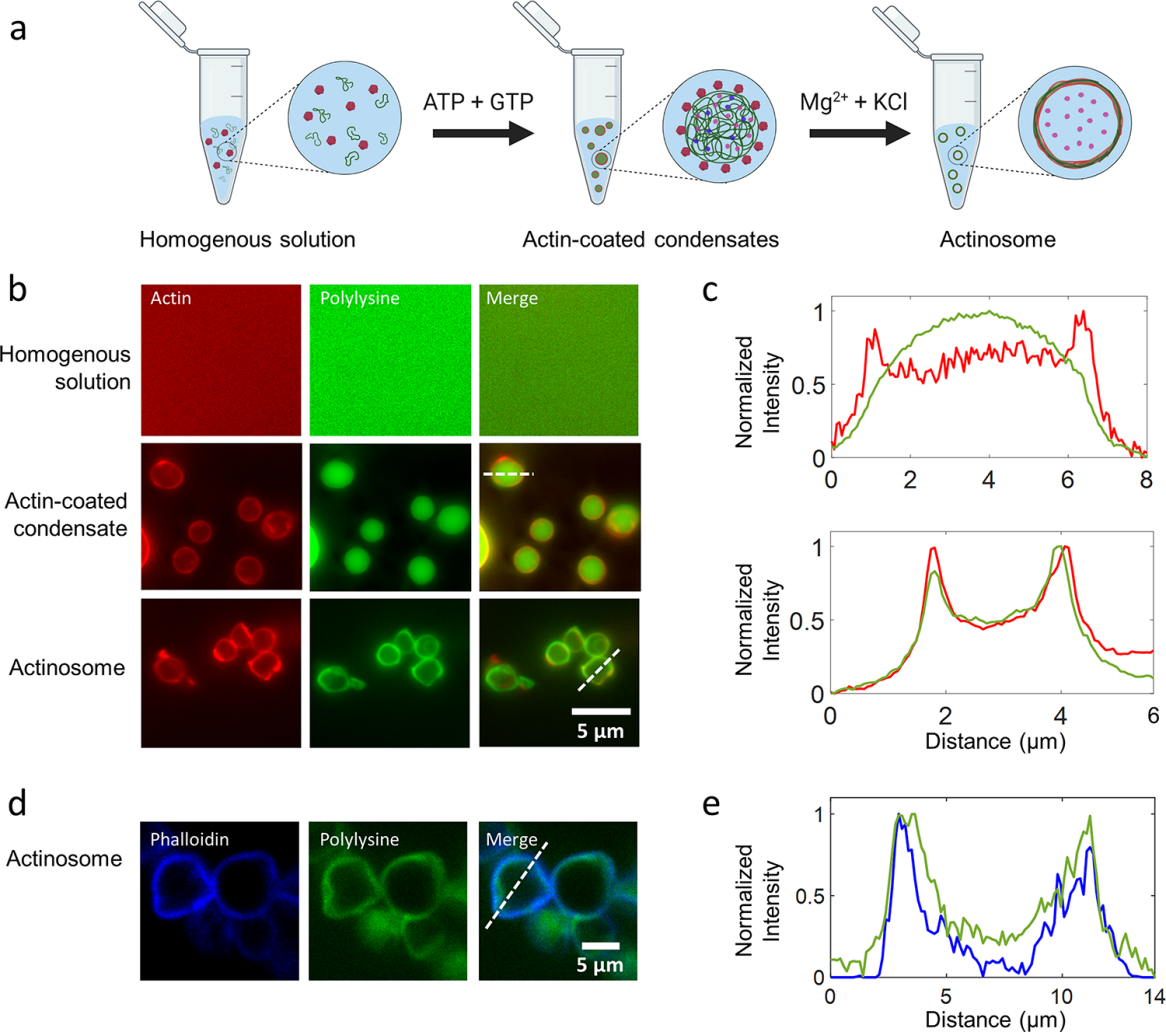
Condensate-templated actinosome formation. (a) Schematic demonstrating stepwise addition of reagents to produce actinosomes. (b) Epifluorescence microscopy images at different stages of actinosome formation. Top: homogeneous mixture of ATTO-532-labeled actin monomers and FITC-labeled polylysine (labeled fraction 10% w/w). Middle: addition of the NTP mixture (GTP + ATP) triggers coacervation, resulting in polylysine/NTP coacervates with actin localized on the surface. Bottom: Mg^2+^ triggers actin polymerization at the expense of ATP hydrolysis, ultimately resulting in coacervate dissolution and formation of a shell made up of actin filaments and polylysine. (c) Line graphs corresponding to the dotted lines in panel (b) showing surface localization of actin on the condensates with polylysine concentrated in the interior (top) and colocalization of the actin and polylysine in actinosomes (bottom). (d) Confocal microscopy images of actinosomes stained with ATTO-594-labeled phalloidin (blue), which selectively binds to actin filaments; FITC-labeled polylysine (labeled fraction 10% w/w) is visualized in green. (e) Line graphs corresponding to the dotted lines in panel (d) showing surface localization of phalloidin-stained actin filaments (blue) along with polylysine (green).

We started with the idea of using membraneless condensates as templates to coat a biomaterial and subsequently dissolve the inner condensate to form a stable container (Figure 1a). We aimed to bring about the structural and chemical transformation of the condensate by coupling a biochemical reaction, ideally carried out by the coated biomaterial itself. Complex coacervates made up of positively charged polypeptides (polylysine, polyK; polyarginine, polyR) and negatively charged NTPs (adenosine triphosphate) are widely used model systems.^34^ With NTPs (ATP and GTP in particular) also being the common energy currency for a wide variety of biochemical reactions, we hypothesized that polyK/NTP would be a good starting point for our experiments. For a fixed amount of polyK (5 mg/mL; molar charge concentration ∼34 mM, assuming all lysine residues are charged and available; average molecular weight per residue 146.19 Da), we determined the optimal concentration of NTPs to attain maximum partitioning in the coacervate phase (Supporting Figure 1). For all of the experiments shown here, unless specified, polyK and total NTP concentrations were thus kept at 5 mg/mL and 5.4 mM, respectively. Using absorbance-based measurements, we estimated the amount of ATP inside the coacervates to be about 50 mM (in the absence of actin), i.e., about 250 times more concentrated than the dilute phase (Supporting Figure 1); the ATP concentration in the dilute phase was measured to be 0.19 ± 0.02 mM (see Methods for details). Our idea strengthened further when the addition of actin monomers to the system strongly partitioned them at the surface of these coacervates (Figure 1b,c), similar to the observations made with other coacervate systems.^35^ Based on fluorescence measurements, we calculated the partition coefficient of actin at the interface to be significantly higher (5.3 ± 1.3, *n* = 61) compared to its partitioning inside the coacervate (3.2 ± 0.7, *n* = 66). In a similar manner, the partition coefficient for polyK inside the coacervate was determined to be (4.2 ± 0.8, *n* = 62). In addition, we used a salt-deficient buffer, keeping the interfacial tension of the coacervate relatively high.^36^ This also significantly prevented partitioning of actin inside the coacervate compared to the surface as we observed that actin relatively partitioned more inside the coacervates in the presence of salt compared to the coacervate–water interface (Supporting Figure 2). Also, the actin present at the coacervate–water interface polymerized into filaments only in the presence of Mg^2+^ ions, as confirmed by the ATTO-594-phalloidin staining (Supporting Figure 3). We further measured the surface potential of the coacervates through ζ-potential measurements. We found the coacervates to be positively charged (16.9 ± 1.5 mV; Supporting Figure 4), agreeing with previous observations.^37^ Interestingly, we also noted that the surface charge always remained positive irrespective of whether the polyK or ATP was in excess, suggesting accumulation of polyK molecules at the surface. Actin being net negatively charged at neutral pH was thus thought to assemble on the surface through electrostatic interactions.^38^ Indeed, surface charge measurements of actin-coated condensates showed significant reduction in the value of the ζ-potential to 7.8 ± 1.1 mV within minutes (Supporting Figure 5). Along with individual coated coacervates, we do observe connected structures of several condensates, which could be attributed to the lowering of the surface potential.

We triggered actin polymerization by adding a hypertonic buffer containing divalent cations (Mg^2+^) and KCl. This initiated the ATPase activity of actin, leading to a rapid hydrolysis of ATP present in the coacervates and formation of actin filaments on the condensate surface. Phalloidin staining confirmed the formation of actin filaments at the condensate surface (Figure 1d,e). Additionally, the osmolarity shock induced *via* hypertonic buffer conditions initiated an outward flow of polyK from the coacervate toward the periphery where actin filaments are localized. To our pleasant surprise, when using an appropriate ratio of the ATP/GTP mixture, the condensates were subsequently converted into micron-sized quasi-spherical confinements within a matter of minutes. As can be seen from the fluorescence images in Figure 1b, actin and polyK signals completely colocalized at the boundary of the (previously present) condensates, while the polyK signal from the lumen was significantly reduced. We aptly termed these containers actinosomes, where the actin filaments together with polyK polymers formed the container boundary, confining a hollow lumen. We observed a higher partition of actin 8.1 ± 1.4 (*n* = 58) at the interface compared to 4.6 ± 0.7 (*n* = 61) inside the actinosome. The polyK localization also showed a similar trend of higher accumulation at the surface (2.9 ± 0.4; *n* = 59) compared to the interior of the actinosomes (2.0 ± 0.3; *n* = 61). Based on the small increase in the dilute phase intensity, a finite fraction of polyK was assumed to leave the condensates altogether. It is important to note that the combination of hyperosmotic shock and actin polymerization was necessary to form actinosomes. Only hyperosmotic shock or only actin polymerization resulted in actin-coated condensates but no actinosome formation (Supporting Figure 6). The hypertonic conditions likely decreased the interfacial tension and facilitated outward movement of polyK.

### ATP:GTP Ratio is Crucial to Actinosome Formation

**Figure 2:**
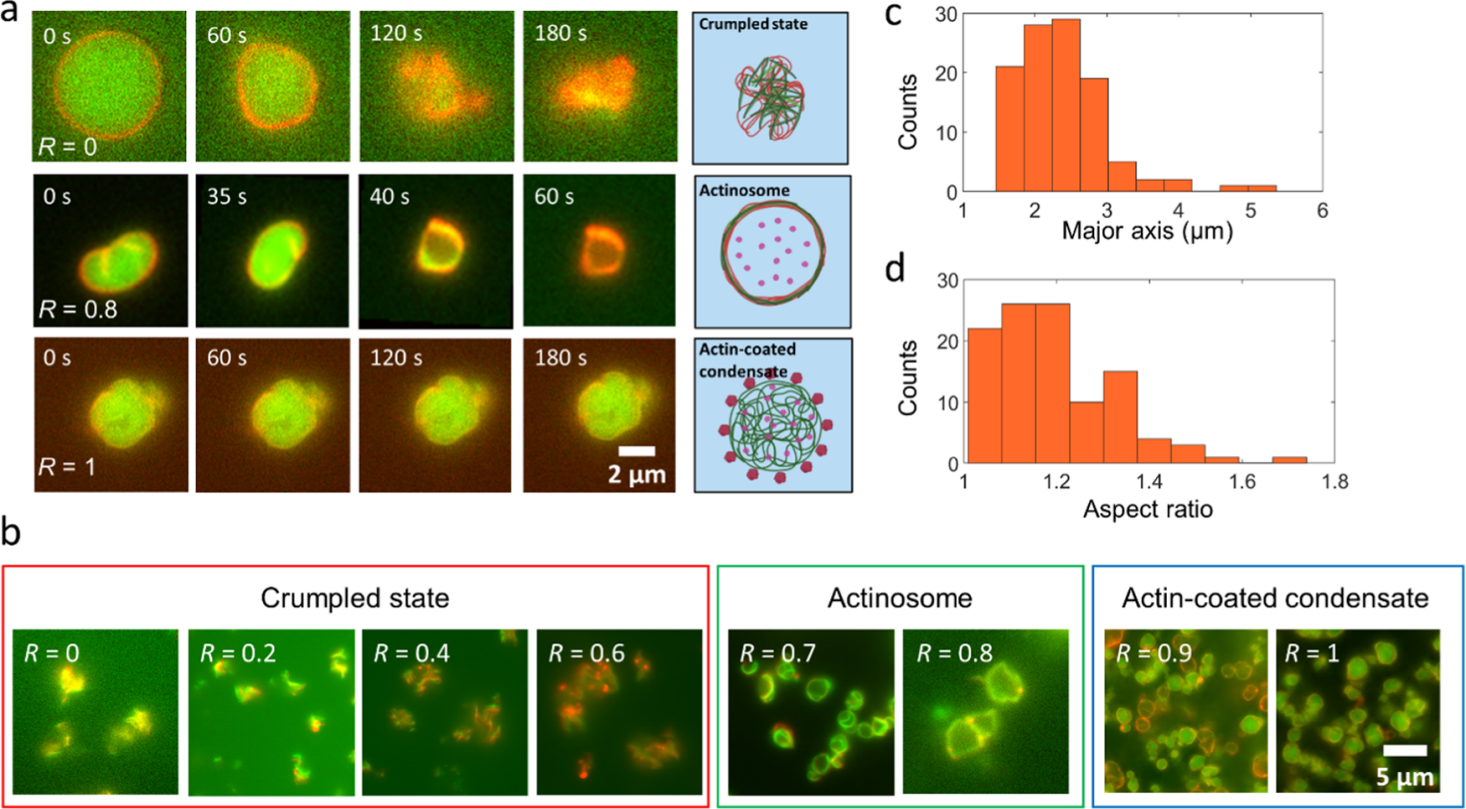
Actinosome formation depends on the ratio of NTPs present in the condensates. (a) Time-lapse images showing the actin-condensate dynamics at different *R* (=[GTP]/([GTP] + [ATP])) values. Low *R* values result in completely crumpled structures, intermediate values form cell-sized actinosomes, while higher *R* values result in stable actin-coated condensates. The inhomogeneous distribution of the actin signal on the actinosome surface is probably due to varying degrees of local actin polymerization. (b) Representative fluorescence images showing three key types of structures formed over the entire range of *R*. Actinosomes are obtained only within a narrow range (0.7 ≤ *R* ≤ 0.8). Lower values (*R* ≤ 0.6) result in crumpled structures, while higher values (*R* ≥ 0.9) lack enough ATP and form stable actin-coated condensates. (c) Frequency histogram showing the size distribution of actinosomes, with a mean size (major axis) of 2.4 ± 0.6 μm (*n* = 107). (d) Frequency histogram showing the ratio of the major axis to the minor axis; the mean value of 1.2 ± 0.1 suggests that actinosomes tend to attain a roughly spherical morphology (*n* = 107). Images were acquired in epifluorescence microscopy.

Since ATP hydrolysis is crucial to coacervate dissolution and subsequent actinosome formation, we studied this further by tuning the ratio of NTPs. We maintained the total concentration of NTPs (GTP + ATP) constant at 5.4 mM and varied the amount of GTP from low to high, which we quantified as *R* = [GTP]/[NTPs]. At *R* = 0, i.e., when using only ATP, the coacervates immediately transitioned from a sphere to a collapsed state, resembling a crumpled structure, like a crumpled sheet of paper (upper panel in Figure 2a, Supporting Movie 1). This phenomenon can be explained as a combination of ATP hydrolysis and colocalization of polyK with actin together with the osmolarity-induced water efflux leading to the buckling of the formed structure. We observed this crumpling prominently for *R* values below 0.6 (Figure 2b). In contrast, actinosomes were efficiently formed for *R* values between 0.7 and 0.8 (Supporting Figure 7). As can be seen in the middle panel in Figure 2a, the polylysine fluorescence rapidly decreased from the lumen and colocalized at the interface along with actin (Supporting Movie 2). Thus, sufficient ATP was present for actin polymerization at the surface, but at the same time, the inert GTP pool maintained enough osmolarity (∼35 mOsm; hydrolyzed ATP possibly contributing further to the value), preventing complete crumpling and resulting in an actinosome with a wrinkled surface. The observed outward flow of polyK toward the periphery was likely promoted by the osmolarity shock induced via the hypertonic buffer. The lack of a coacervate interior, judged by the lack of polyK fluorescence in the lumen but rather its colocalization with actin, strongly suggests the presence of a non-phase-separated aqueous lumen. A *z*-stack of the actinosome makes this clearer, showing colocalization of actin and polyK across the entire structure and showing actin- as well as polyK-depleted lumen (Supporting Movie 3). Owing to the slight crumpling of the shell due to osmotic effects, the formed actinosomes were not perfectly spherical but were quite irregular in shape. We calculated the average size of actinosomes, by approximating them as ellipses, to be 2.4 ± 0.6 μm (major axis ± standard deviation; *n* = 107; Figure 2c). We measured the eccentricity (major axis/minor axis) to quantify their spherical nature. A value of 1.2 ± 0.1 shows that actinosomes remained reasonably spherical (Figure 2d). We observed that not all of the actin-coated condensates converted into coacervates, possibly due to heterogeneity of the actin coating, subsequent inhomogeneous polymerization, and thus different degrees of osmotic shock between different condensates. The actinosome yield (number of actinosomes obtained/total number of actinosomes and actin-condensate structures) was determined to be ∼0.7 (actinosomes, *n* = 198; actin-coated coacervates, *n* = 88). We also observed that the actinosomes tend to form clusters, i.e., two or more actinosomes sticking to each other (structures containing actin-coated condensates were excluded for analysis). Quantitative analysis showed that about 25% of the actinosomes remained in the individual isolated state, whereas the remaining 75% tended to form clusters of 2–5 actinosomes (Supporting Figure 8). At *R* values above 0.9, we observed a mixed population of both actinosomes and coacervates coated with actin (Figure 2b). At *R* = 1, we observed only actin-coated condensates (lower panel in Figure 2a; Supporting Movie 4). With not enough ATP to bring about actin polymerization and coacervate dissolution, these coacervates remained stable and did not show any morphological changes over time. Thus, the ratio of GTP to ATP is crucial to actinosome formation.

We also checked the effect of the nature of the polypeptide on actinosome formation, where we used poly-l-arginine (polyR) to form the coacervates. While we obtained actin-coated condensates, we did not see complete crumpling when using only ATP or a container formation when using a mix of ATP and GTP; this trend continued even after doubling the salt concentrations (Supporting Figure 9). This is possibly due to the significantly higher (100-fold) viscosity and surface tension (5.8-fold) of the polyR-containing droplets as compared to polyK-containing ones,^39^ potentially preventing the rapid exchange of material across the interface and insufficient ATP diffusion to the surface.

### Actinosomes Are Hollow and Porous Containers

**Figure 3:**
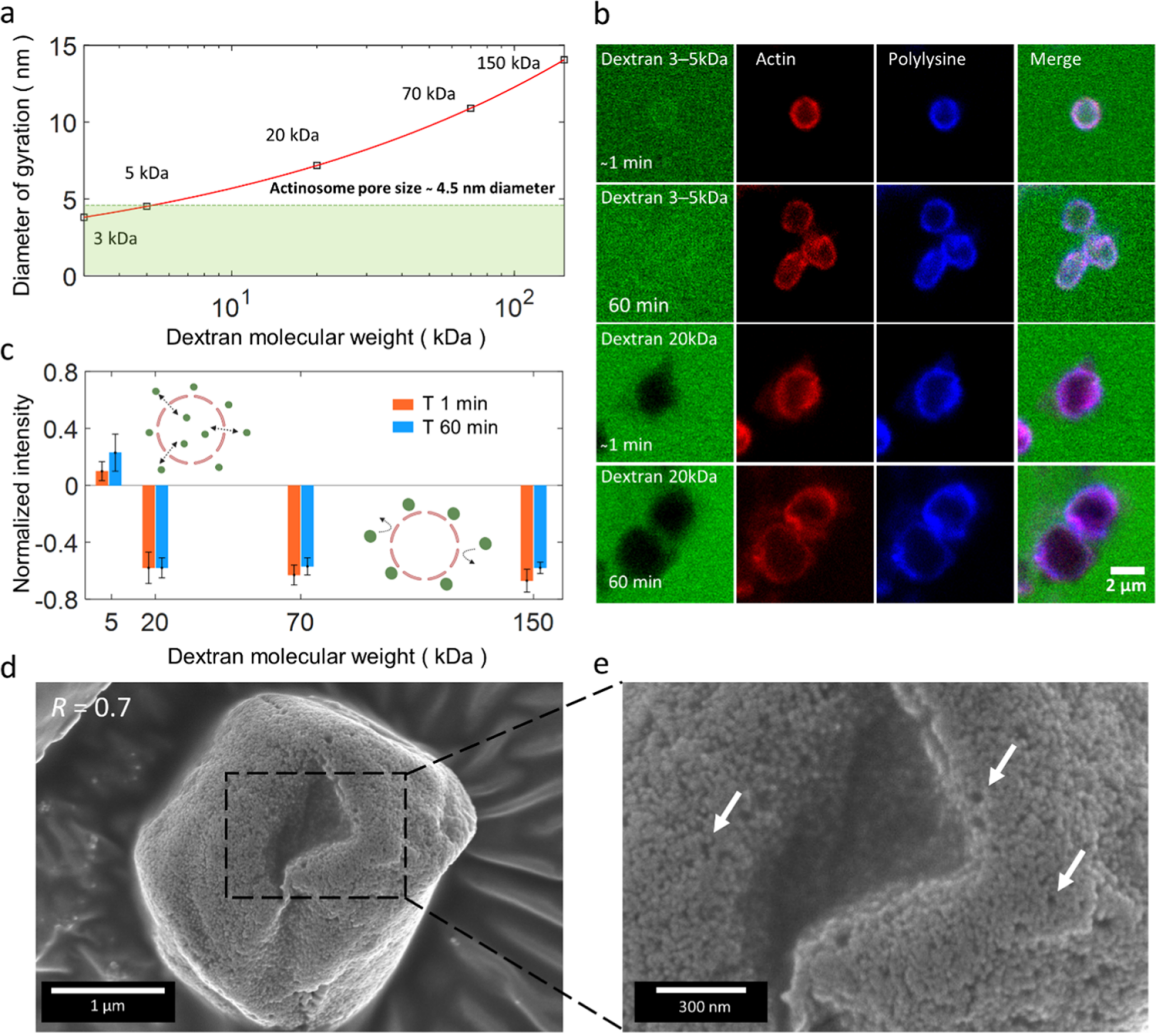
Actinosomes are porous and permeable to small molecules. (a) Diameter of gyration (*D*_g_) of dextran molecules as a function of their molecular weights (*M*). The red line follows the equation *D*_g_ = 2.64 × *M*^0.33^. (b) Confocal images showing the permeability of actinosomes (*R* = 0.7) to dextran molecules of different sizes, immediately (*t*_0_) and 1 h (*t*_60_) after incubation. Low-molecular-weight dextran (3–5 kDa) readily diffuses inside actinosomes, whereas high-molecular-weight dextran (20 kDa) is excluded from the actinosome. (c) Graph showing the normalized intensity (*I*_inside_ – *I*_outside_)/*I*_outside_ of FITC-dextran at *t*_0_ (red) and *t*_60_ (blue). Positive values indicate dextran diffusion into the actinosomes, while negative values indicate impermeability to dextran. Error bars indicate standard deviations. (d) Scanning electron microscopy images of actinosomes (*R* = 0.7) appear as slightly crumpled spheres, similar to fluorescence images. (e) A zoom-in reveals a rough, unstructured, porous surface. Several sub-μm-sized pores are clearly visible and indicated with arrows. Error bars indicate standard deviations.

A mesh of polylysine and actin filaments comprises the actinosome surface. To characterize the surface permeability, we tested the diffusion-driven influx of dextran molecules of a variety of sizes into the actinosome lumen. We incubated premade actinosomes (*R* = 0.8) with FITC-labeled dextran solution (concentration kept constant at 4 μM for all of the experiments) of different molecular weights (*M*), viz., 3–5, 20, 70, and 150 kDa corresponding to the diameter of gyration (*D*_g_) values of 3.81–4.52, 7.18, 10.90 and 14.05 nm, respectively^40^ (Figure 3a). The low-molecular-weight dextran molecules (3–5 kDa) immediately (*t*_0_, corresponding to approximately within a minute after addition of dextran) permeated inside the actinosomes (Figure 3b). On the contrary, actinosomes were not permeable to any of the higher-molecular-weight dextran molecules (>20 kDa) for the entire duration of 60 min. Figure 3b shows the exclusion of 20 kDa dextran molecules from actinosomes, while Supporting Figure 10 shows images corresponding to dextran assays corresponding to 70 and 150 kDa. To characterize the permeability, we measured the FITC-dextran signal inside (*I*_inside_) and outside (*I*_outside_) actinosomes and calculated the normalized intensity as (*I*_inside_ – *I*_outside_)/*I*_outside_. We analyzed this for images taken immediately (*t*_0_) as well as after one hour (*t*_60_). The positive normalized intensity for 5 kDa (*t*_0_: 0.12 ± 0.07, *n* = 19; *t*_60_: 0.23 ± 0.13, *n* = 20) dextran indicates influx of dextran inside actinosomes (Figure 3c). On the contrary, negative normalized intensity for 20 kDa (*t*_0_: −0.58 ± 0.11, *n* = 23; *t*_60_: −0.58 ± 0.07, *n* = 24), 70 kDa (*t*_0_: −0.63 ± 0.07, *n* = 24; *t*_60_: −0.57 ± 0.06, *n* = 30), and 150 kDa (*t*_0_: −0.67± 0.08, *n* = 25; *t*_60_: −0.58 ± 0.04, *n* = 35) clearly indicates exclusion of dextran inside the actinosomes. Based on the above analysis, we conclude that actinosomes are porous containers with a pore size of ∼5 nm and definitely below 7 nm (Figure 3a,b).

After determining the pore size of actinosomes, we moved our attention to the topological characterization of actinosomes. For this, we performed scanning electron microscopy (SEM) on actinosomes to visualize the detailed surface morphology. We dried and sputtered samples of actinosomes (*R* = 0.74), crumpled condensates (*R* = 0.55), and actin-coated condensates (*R* = 0.92) for visualization (see Methods for details). The actinosome surface revealed a rough shell (Figure 3d) in which submicron-sized pores on the order of 0.02–0.05 μm in diameter were visible (Figure 3e), supporting the previously described permeable interface allowing migration of molecules across the rigid shell. In addition to this, visualizing a broken actinosome revealed a hollowness in the interior of the actinosome (Supporting Figure 11a,b). The shells appeared rigid, given that they survived the vacuum-drying process. On the other hand, the surface of polylysine/NTP coacervates (*R* = 0.92) with actin localized on the surface was relatively smooth and did not show any of the above-mentioned features (Supporting Figure 11c). At high ATP concentration (*R* = 0.55), crumpled structures were observed (Supporting Figure 11d), corroborating with the fluorescence images obtained before.

### Actinosomes Efficiently Encapsulate Biomolecules and Carry Out Complex Biochemical Reactions

**Figure 4:**
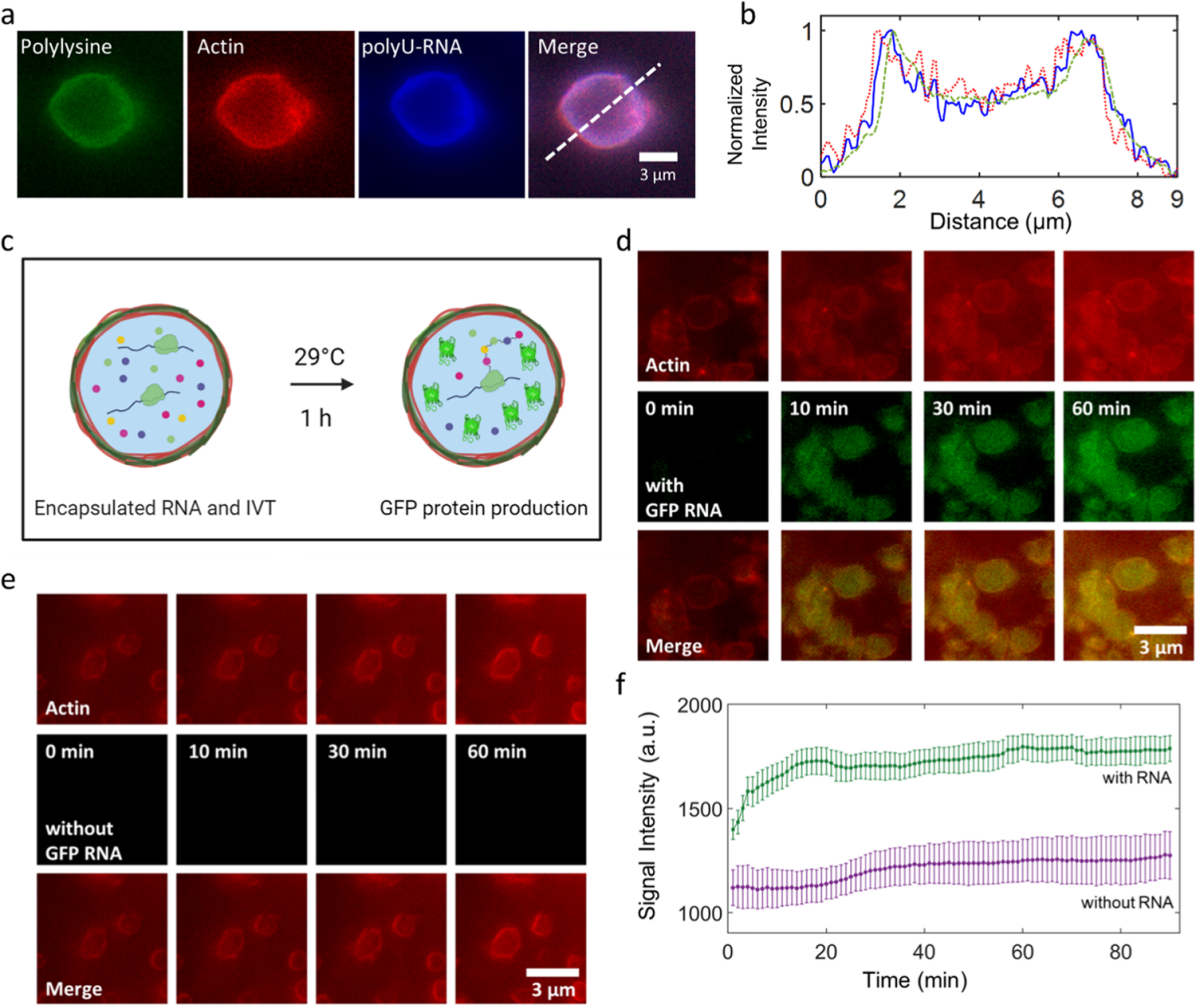
Actinosomes as protein-producing bioreactors. (a) Encapsulation of Cy5-labeled RNA (1.25 mM, polyU 20-mer) encapsulated inside the actinosomes. (b) Line graph corresponding to the dotted line in panel (a) showing the localization of polyU-RNA (solid line, blue) near the actinosome border. Actin (dotted line, red) and polylysine (dashed line, green) profiles are also shown. (c) Schematic illustrating actinosomes encapsulating an *in vitro* translation machinery along with GFP-mRNA (left). Upon incubation, GFP-mRNA inside the actinosomes produces active GFP protein that remains encapsulated. (d) Expression of GFP inside actinosomes by encapsulation of GFP-encoding mRNA and a cell-free *in vitro* translation machinery (IVT). As can be seen, GFP fluorescence (green) increases over the course of an hour inside actinosomes, while the background remains dark, indicating the protein expression is carried out predominantly inside the containers. (e) Negative control (no GFP-mRNA but translation machinery is still present) showing no increase in fluorescence over the same duration. (f) Analysis of GFP expression inside actinosomes (*n* = 11) showed an initial steep increase before gradually reaching a plateau over the course of an hour. Analysis of actinosomes (*n* = 6) lacking GFP-encoding RNA but with encapsulated IVT had a significantly lower and a relatively constant signal intensity over the same period. We note that the *t* = 0 min corresponds to roughly 5 min after the reaction had started; the delay is caused due to technical limitations like adjusting the focus and selecting an optimal field-of-view. Error bars indicate standard deviations. Images were acquired in epifluorescence microscopy.

With the intention to confine molecules within the containers, we encapsulated RNA, given its central importance in the cellular metabolism, and added it to the starting mixture of polyK and actin. We found that fluorescently (Cy5) labeled RNA (a 20-mer polyU) could be efficiently encapsulated inside the actinosomes (Figure 4a). The partition coefficient of RNA was 4.0 ± 1.0 (*n* = 10) in the lumen of actinosomes and higher 7.0 ± 1.3 (*n* = 10) near the inner surface of actinosomes. This can be further seen by plotting a line profile of the fluorescent intensity across the actinosome showing colocalization of RNA and the polylysine signal (Figure 4b). This is likely due to the electrostatic interaction between negatively charged RNA and positively charged polyK polymers, leading to a nonhomogeneous RNA distribution. We did not see any appreciable leakage of RNA fluorescence outside the actinosomes over a course of more than an hour.

One of the trademark properties of coacervates is their ability to selectively sequester biomolecules^41^ within them, often up to orders of magnitude higher than the surroundings.^42^ Coacervates also provide a distinct microenvironment that can differ from the dilute phase like the concentration of metal ions (such as Mg^2+^).^25^ Thus, condensate droplets acting as the initial scaffolds for actinosomes provide an excellent opportunity to preload the actinosomes with components of interest. We tested this strategy by sequestering a cell-free protein translation machinery (rabbit reticulocyte lysate) along with single-stranded, capped, and tailed mRNA encoding the enhanced green fluorescence protein (EGFP) inside the coacervate droplets (Figure 4c). This was done by adding the necessary components to the initial mixture prior to condensate formation. Upon subsequent actinosome formation, based on our pore size measurements, we expected the large biomolecules involved in the cell-free expression machinery to remain encapsulated within actinosomes. We then incubated the actinosomes at 29 °C and monitored the GFP expression in real time (Figure 4d, Supporting Movie 5). As can be seen, fluorescence in the GFP channel steadily increased over the course of an hour, with the protein expression evident as early as in the first few minutes. We attribute the quick maturation of GFP protein to the usage of an EGFP-mRNA construct and the cell-free translation machinery.^43^ The protein expression taking place inside the actinosomes also suggests that the RNA is localized on the inner surface of the actinosomes (Figure 4a) and not on the outer one because otherwise the expressed proteins would have simply diffused away. Actinosomes encapsulating the *in vitro* translation machinery without the GFP-encoding mRNA showed no signal in the GFP channel over a similar time course (Figure 4e). The GFP expression was further analyzed by measuring the fluorescence signal intensity inside the actinosomes. A steady increase in intensity (*n* = 11) was observed for the first 20 min, which later plateaued (Figure 4f). Actinosomes without the GFP-encoding mRNA but still carrying the *in vitro* translation machinery showed no increase in the signal intensity (*n* = 6). The ease of encapsulating a complex machinery without needing any sophisticated setup and conducting biochemical reactions makes actinosomes suitable bioreactors.

## Discussion

**Figure 5:**
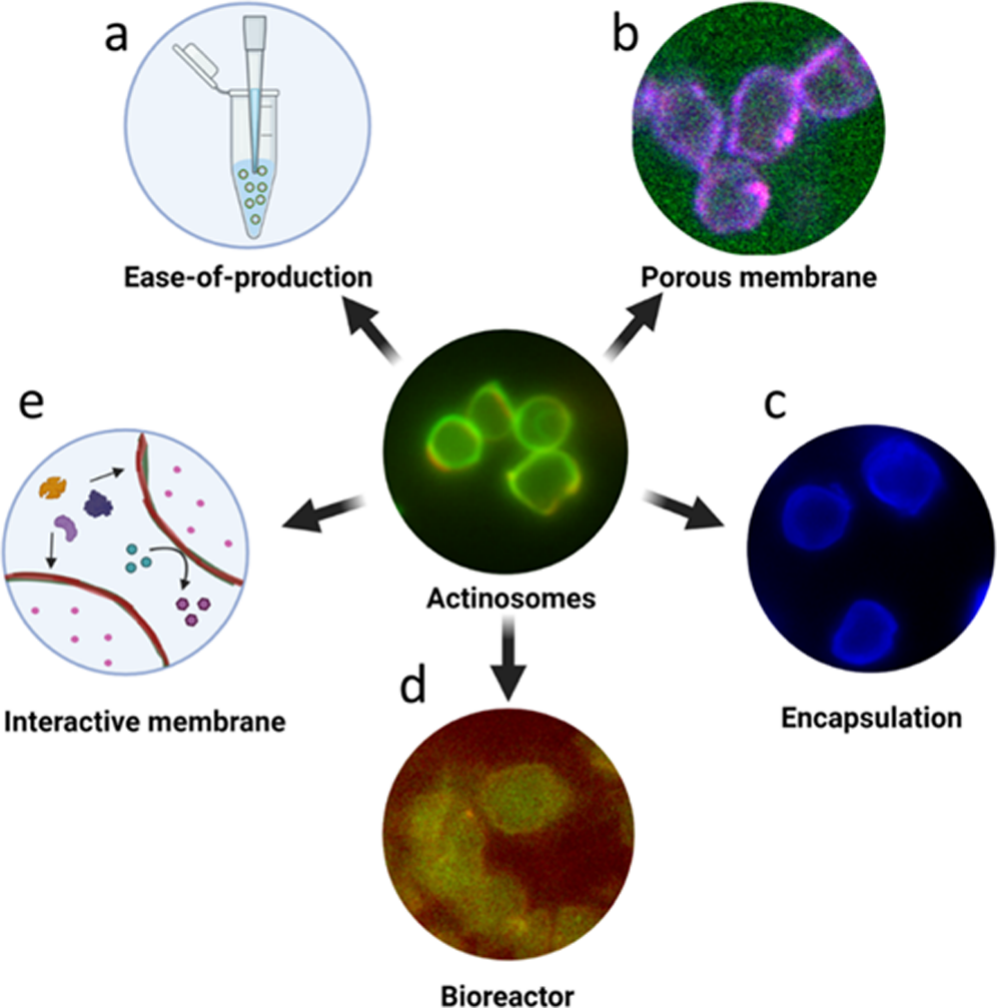
Salient feature of actinosomes. Actinosomes are synthetic confinements with a boundary made of polyK and actin filaments. Several properties make them potentially useful containers for synthetic cell research. (a) They are easy to produce without the need for any sophisticated setups. (b) They have a permeable surface allowing small biomolecules to pass through. (c) They can efficiently encapsulate biomolecules owing to the inherent sequestration capacity of condensates. (d) They have the capacity to act as bioreactors to conduct complex reactions like protein translation. (e) The actin-based boundary opens up the possibility of having an interactive membrane for recruiting other proteins, designing signaling cascades, and forming multicellular assemblies.

In this paper, we have presented actinosomes: three-dimensional, cell-sized confinements with a boundary made up of polylysine polymers and actin filaments (Figure 5). The unstructured and porous proteinaceous shell provides a stable boundary, allowing biochemical reactions to take place inside the container. Actinosomes are quick and easy to make, especially compared to other containers such as liposomes and proteinosomes, which are currently used to form synthetic cells. Furthermore, the use of condensates as templates helps in encapsulation of a wide variety of biomolecules owing to their intrinsic ability to get sequestered in the condensate phase.

The current extensive use of microfluidic techniques to generate highly monodispersed containers and achieve efficient encapsulation also adds a significant amount of complexity to the production techniques.^44,45^ An easy and robust process to produce microconfinements can thus be useful for specific purposes, especially in resource-limited conditions. We have shown that actinosomes are relatively straightforward to produce and can carry out complex biochemical reactions. While they might not be suitable for certain cellular features like growth and division, they are certainly appealing to be used as chemical nanofactories and for studying the effect of confinement on biochemical processes. With regard to monodispersed samples, actinosomes were found to be surprisingly uniform in size (average major axis: 2.4 ± 0.6 μm). We think this is due to nucleation, and subsequently coacervation, taking place homogenously throughout the solution and the droplets getting immediately stabilized by actin, further preventing their coalescence and leading to a fairly homogeneous size distribution. Changing the relative concentrations of principal components, especially actin, might allow further tuning of the size. With regard to its limitations, we do note that not all of the molecules can be naturally sequestered in condensates.^46,47^ Also, within actinosomes, small molecules are prone to diffusing across the boundary over time. Additionally, based on the coacervate species, the partition coefficient can vary ranging from ∼1 to >100, suggesting not all biomolecules concentrate equally.^48^ Finally, actinosomes have a tendency to form clusters and sometimes aggregate, which needs to be tackled to make them more suitable for systematic biological applications.

We propose the following mechanism for actinosome formation. We begin with an initial homogeneous solution of actin, polylysine, and other biomolecules that one wishes to sequester inside actinosomes. Upon addition of the NTP mixture (ATP + GTP) to the solution, complex coacervation is induced between polylysine and NTPs, forming coacervate droplets. Actin preferentially decorates the surface of the condensates, aided partially by the electrostatic interactions between the net negatively charged actin protein and positively charged condensates and the viscous nature of the coacervate due to the absence of salt. Other biomolecules present (ones that are to be encapsulated inside) are likely to get partitioned inside, or alternatively at the surface of, the condensate. It is important to note that at this point, actin stays in the monomeric form as there are no Mg^2+^ ions present in the system, which are essential for polymerization. Addition of a salt-containing buffer (Mg^2+^ and KCl) triggers rapid actin polymerization at the expense of the ATP that is highly concentrated (∼50 mM) in the condensates. Conversion of ATP into ADP and Pi (inorganic phosphate) leads to dynamic changes in the coacervate composition. However, polylysine polymers cannot readily diffuse outside and remain entangled within actin filaments to form an unstructured shell at the interface. This process continues until a majority of the polylysine is colocalized with the actin at the surface. This eventually leads to dissolution of the original condensate droplet to ultimately form a microcontainer, comprising an aqueous lumen surrounded by a proteinaceous shell.

We observe that the addition of a monovalent salt (KCl) plays an important role in actinosome formation. It weakens the electrostatic attractions between the coacervate components and possibly facilitates ATP consumption by actin. Furthermore, addition of KCl presents a hyperosmotic shock that seems to result in a water flux out of the forming actinosomes and induces an outward movement of polylysine molecules, allowing them to get entangled with actin filaments. This logic is consistent with the different scenarios we observe as we change *R*. In the case of a low-enough GTP content (*R* ≤ 0.6), the hyperosmotic shock (Δ*c* ∼200 mosm; Δ*P* = Δ*cRT* = 0.5 MPa) is too strong, resulting in significant loss of the water content from the condensate, eventually resulting in a crumpled state lacking structural integrity. At intermediate GTP contents (*R* = 0.7–0.8), while there is efflux of water, the NTP concentration (∼50 mM) is enough to sustain the osmotic pressure difference until the salt equilibrates. At high GTP contents (*R* ≥ 0.9), there are no significant morphological changes as the actin does not polymerize readily due to lack of enough ATP and thus condensate components do not really change. Thus, actin polymerization at the expense of ATP inside the condensates in combination with salt flux together drives actinosome formation.

In conclusion, actinosomes are a novel addition as synthetic cell containers with useful properties. They are easy to produce and require only basic lab equipment and commercially available proteins (Figure 5a). They have a porous membrane, with a pore size of ∼5 nm, allowing easy transport of small biomolecules but retaining larger biomolecules (Figure 5b). As a result, they can efficiently encapsulate macromolecules, especially negatively charged polymers like RNA (Figure 5c). They can further carry out biochemical reactions by simply adding all of the required components in the initial mixture. We demonstrated this by encapsulating the entire translation machinery, which consists of complex biomolecules including enzymes and tRNA molecules (Figure 5d). Lastly, actin-based membranes present interesting opportunities to functionalize these containers (Figure 5e). For example, actin can interact with numerous actin-binding proteins to initiate specific reactions at the interface. This can be used in forming communicative networks within a population or even physically connect the containers to form multicomponent, tissue-like structures. Such functionalities together with their highly biocompatible nature may allow actinosomes to interact with living cells and form hybrid interfaces. Further systematic research in these directions will reveal the true potential of these proteinaceous confinements and their use as scaffolds for synthetic cells.

## Materials and Methods

### Chemicals and Proteins

Unlabeled poly-l-lysine (molecular weight (*M*_W_) 15–30 kDa) and fluorescently labeled FITC-poly-l-lysine (*M*_W_ 15–-30 kDa) were purchased from Sigma-Aldrich. Individual nucleotides (ATP and GTP) were purchased from Thermo Scientific. Cy5-labeled polylysine (*M*_W_ 25 kDa) was purchased from Nanocs Inc. Actin (rabbit skeletal muscle α actin), fluorescently labeled ATT0 532-actin (rabbit skeletal muscle α actin), and ATT0 594-actin were purchased from HYPERMOL in the form of lyophilized powders. The composition of the reconstitution buffer to dissolve actin monomers was 2 mM Tris (pH 8.0), 0.4 mM ATP, 0.1 mM CaCl_2_, and 0.01 mM dithiothereitol. The end composition of the actin polymerization buffer was 0.01 M imidazole pH 7.4, 0.1 M KCl, and 2 mM MgCl_2_. Fluorescently labeled ATTO-594-phalloidin was purchased from HYPERMOL (Cat. No. C8815-01). For permeability experiments, we used various FITC-labeled dextran solutions: *M*_W_ 3–5 kDa (Sigma, Cat. No. FD4; mol FITC/mol glucose = 0.001–0.02), FITC-labeled dextran M_W_ 20 kDa (Sigma, Cat. No. FD20S; mol FITC/mol glucose = 0.003-0.02), FITC-labeled dextran *M*_W_ 70 kDa (Sigma, Cat. No. 46945; mol FITC/mol glucose = 0.004), and FITC-labeled dextran *M*_W_ 150 kDa (Sigma, Cat. No. 46946; mol FITC/mol glucose = 0.004) to actinosome. Polyvinyl alcohol (PVA), molecular weight 30,000–70,000, 87–90% hydrolyzed, was purchased from Sigma-Aldrich.

### Actinosome Synthesis

The process of making actinosomes can be summed up in three distinct steps: (1) preparing the actin–polylysine mixture; (2) forming coacervates with coated actin; (3) and actin polymerization and coacervate dissolution. Step 1: Monomeric actin and polylysine were reconstituted in the actin reconstitution buffer, with final concentrations of 3 μM and 5.05 mg/mL, respectively. The pH 8 of the buffer is crucial for monomeric actin stability. Additionally, it keeps the polylysine polymers positively charged. For microscopic visualization, the sample was doped with 10% fluorescently labeled actin (0.3 μM) and 1% FITC-poly-l-lysine (0.05 mg/mL). Step 2: To trigger coacervation, 5 mM NTP mixture (e.g., 1.25 mM GTP and 3.75 mM ATP) was added to the solution and gently pipetted to mix thoroughly. Step 3: To make actinosomes, actin polymerization buffer was added to the actin-coated coacervate solution. The sample was vortexed briefly to ensure sufficient mixing, followed by a short spin (1000 rpm for 5–10 s) to remove any large aggregates. The last step significantly increased the yield of separated (not connected in clusters) actinosomes.

### ζ-Potential Measurements

The net surface charge of the coacervate was determined by measuring the ζ-potential at 25 °C using the Malvern Zetasizer Nano instrument. The sample was diluted 1:20 and gently mixed prior to measurements. The ζ-potential for each sample was determined by taking the average measurement of three independent samples, where each measurement is the average of five readings from the same sample.

### ATP Concentration Measurements

To determine the NTP concentration required to obtain the maximum amount of the condensate phase for a given polylysine concentration, we prepared buffered solutions (2 mM Tris (pH 7.4), 100 mM KCl, and 2 mM MgCl_2_) containing different concentrations of ATP (from 1.25 to 25 mM) while keeping the polylysine concentration constant at 5 mg/mL. The solution was incubated at room temperature for 15 min to equilibrate. The condensed phase was separated from the dilute phase by centrifugation at 10,000 rpm for 5 min. The concentration of the free ATP in the dilute phase was evaluated by measuring its absorbance at 259 nm using the molar extinction coefficient of ATP (15,400 M^–1^ cm^–1^) using UV–vis absorption spectroscopy (NanoDrop 2000/2000c spectrophotometer, Thermo Scientific). The concentration of ATP inside the coacervates was calculated as *c*_dense_ = (*c* – *c*_dilute_*f*)/(1 – *f*), where *c*_dense_ and *c*_dilute_ are the ATP concentrations in dense and dilute phases, respectively, and *f* is the volume fraction of the dilute phase. Concentration in the dilute phase, *c*_dilute_, was measured by absorbance as stated above. The fraction of the dilute phase, *f*, was estimated to be 0.9 by carefully removing the supernatant after centrifugation without disturbing the dense phase. For example, from a 40 μL sample, we estimated 36 μL to be the dilute phase.

### Fluorescence Microscopy

The samples were imaged on a Nikon-Ti2-Eclipse inverted fluorescence microscope, equipped with a pE-300^ultra^ illumination system, using a Nikon Plan Apo 100*x*/1.45 NA oil objective. FITC-polyK and GFP expressions were detected using a 482/35 nm excitation filter and a 536/40 nm emission filter (Semrock). Actin-ATTO-532 was detected using a 543/22 nm excitation filter and a 593/40 nm emission filter (Semrock). Actin-ATTO-594 was detected using a 628/40-25 nm excitation filter and a 692/40-25 nm emission filter (Semrock). The samples were illuminated at 2–5% laser intensity, and time-lapse images were acquired using a Prime BSI Express sCMOS camera. Exposure time was usually 10–20 ms except for GFP visualization, when it was increased to 50–100 ms. The dextran influx assay was visualized using a confocal microscopy setup using laser of wavelengths 488, 561, and 640 nm for FITC-dextran, ATTO-594-labeled actin, and Cy5-labeled polylysine, respectively. For the phalloidin assay, actinosome (*R* = 0.8) was incubated for 1 h with 5 μL of phalloidin (stock prepared using manufacturer’s protocol) and was visualized using a confocal microscopy setup 488 and 561 nm for FITC-labeled polylysine and ATTO-594-labeled phalloidin, respectively.

### Microscopy Setup

Samples were visualized in small chambers made of poly(dimethylsiloxane) (PDMS) and glass slides (Supporting Figure 12). The device was fabricated as follows. PDMS and the curing agent were mixed at a mass ratio of 10:1, and the air bubbles trapped during mixing were removed by desiccating in a vacuum desiccator. The mixture was poured on a silicon wafer (75 mm in diameter) and cured by baking at 80 °C for 4 h. Holes of 5 mm diameter were punched in the PDMS block using a biopsy punch. The PDMS and a clean glass slide (#1.5, VWR International) were plasma-treated and bonded together using a plasma cleaner (Harrick Plasma PDC-32G).

To minimize coacervates wetting the surface, the glass slide was coated with 5% w/v poly(vinyl alcohol) immediately after plasma bonding. The PVA solution was incubated for 10 min in the wells and discarded. The wells were rinsed with Milli-Q water to remove uncoated PVA. The devices were baked at incubated 120 °C for 10 min to heat-immobilize the PVA polymers on the surface. The device was ready to use for microscopic visualization once cooled down.

### SEM Microscopy

The surface of actinosomes was analyzed by scanning electron microscopy. Actinosomes were prepared and vacuum-dried at room temperature on electrically conductive carbon adhesive discs mounted on a metal stub. The dried samples were sputter-coated with Tungsten (to obtain a thin film of ∼12 nm). The acquired images were taken at approximately 65,000*x*–85,000*x* magnification at 2–3 kV accelerating voltage and 13 pA current.

### RNA Expression in Actinosomes

A capped and tailed messenger RNA (mRNA) template, encoding an enhanced green fluorescent protein, was synthesized from a linearized double-stranded DNA (Supporting Figure 13) using the HiScribe T7 ARCA mRNA kit (New England Biolabs, Ipswich, MA). The synthesized mRNA was purified using the Monarch RNA cleanup kit (New England Biolabs, Ipswich, MA), thereby removing the template DNA. GFP-encoding mRNA (final concentration 50 ng/μL) along with the 37.5% v/v *in vitro* translation machinery Flexi Rabbit Reticulocyte Lysate System (Promega) was added along with actin and polylysine in step 1, prior to the addition of NTPs. This strategy allows efficient encapsulation of GFP-mRNA and translation machinery inside the actinosomes. Real-time expression of GFP was monitored by incubating actinosomes at 29 °C using the Okolab heating stage.

### Image Analysis

Since the morphology of actinosomes is close to that of a sphere, the size of the actinosomes was determined by fitting an ellipse using the Fitting Elipse function in Fiji. The obtained major and minor axes were used to determine the aspect ratio. For calculating the partition coefficient, the mean fluorescent intensity of actin, polylysine, or RNA inside or at the surface of the coacervates (*I*_dense_) was measured for several coacervates, along with the mean fluorescent intensity outside the coacervates (*I*_dilute_). The background intensity, *I*_bg_, was measured outside the sample. The corresponding partition coefficient was then calculated as .

## Supporting Information Available

Crumpling of actin-coated condensate (AVI)
Efflux of polylysine during actinosome formation (AVI)
Z-stack of an actinosome (AVI)
Stable actin-coated condensate (AVI)
Expression of green fluorescent protein inside actinosomes (AVI)
Additional experimental results including graphs of ζ-potential of actin-coated coacervates (Figures S1, S4, and S5); fluorescence microscopy images of control experiments (Figures S2, S3, S6, and S9); full field of view of actinosomes (Figure S7); actinosome cluster analysis (Figure S8); confocal images of the dextran exclusion assay (Figure S10); scanning electron microscope images of actinosomes at varying NTP ratios (Figure S11); experimental setup (Figure S12); DNA sequence of green fluorescent protein (Figure S13) (PDF)

## Supplementary Material

sb2c00290_si_001.avi

sb2c00290_si_002.avi

sb2c00290_si_003.avi

sb2c00290_si_004.avi

sb2c00290_si_005.avi
