# Supplementary material for: Actinosomes: Condensate-Templated Containers for Engineering Synthetic Cells
Source: ACS Synth Biol. 2022 Aug 10;11(8):2869–79. doi: 10.1021/acssynbio.2c00290 (PMC9396703; doi:10.1021/acssynbio.2c00290)
Supplement: Supplementary file 6 — sb2c00290_si_006.pdf [file sb2c00290_si_006.pdf]

Actinosomes: condensate-templated containers for engineering synthetic cells

Ketan A. Ganar, Liza Leijten, Siddharth Deshpande*

Laboratory of Physical Chemistry and Soft Matter, Wageningen University and Research, Stippeneng 4, 6708 WE Wageningen, The Netherlands

Supplementary figures

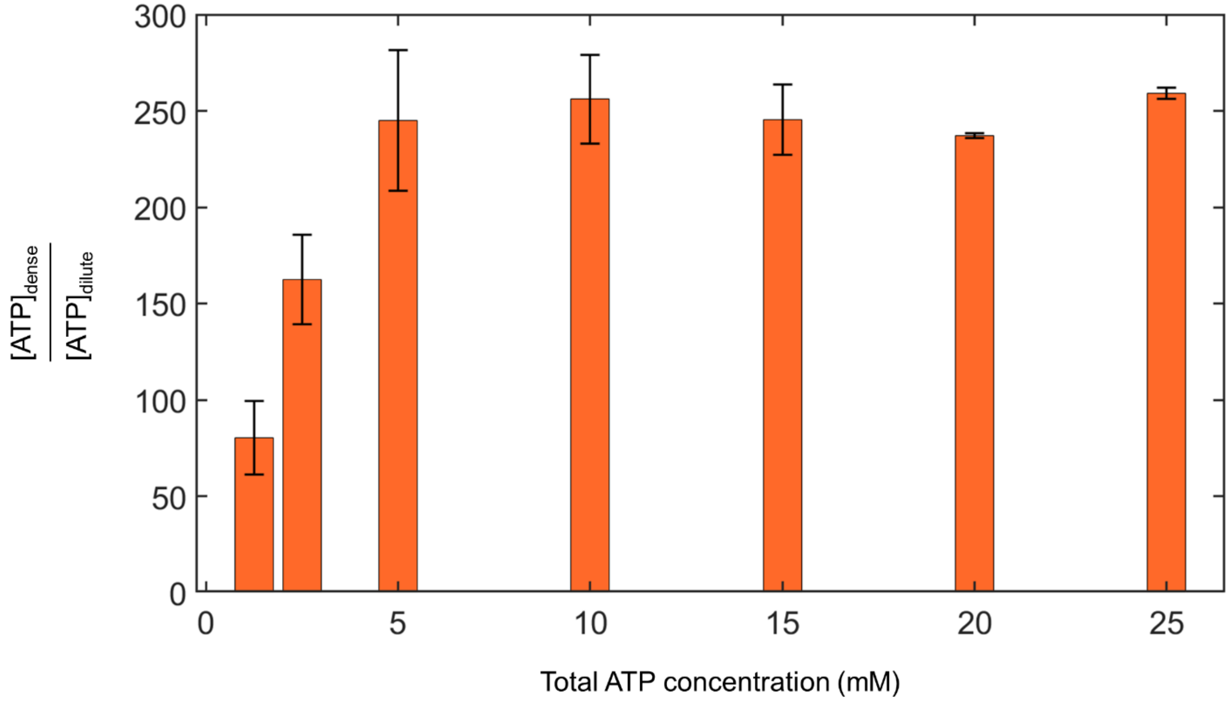

Supplementary Figure 1. Determining the NTP concentration that leads to optimal coacervation with polyK. Amount of ATP partitioned inside the dense phase at constant polylysine concentration of 5 mg/ml. The maximum partitioning (around 250-fold) was observed for a total ATP concentration above 5.0 mM (n = 3 experimental repeats; error bars indicate standard deviations; see Methods for details).

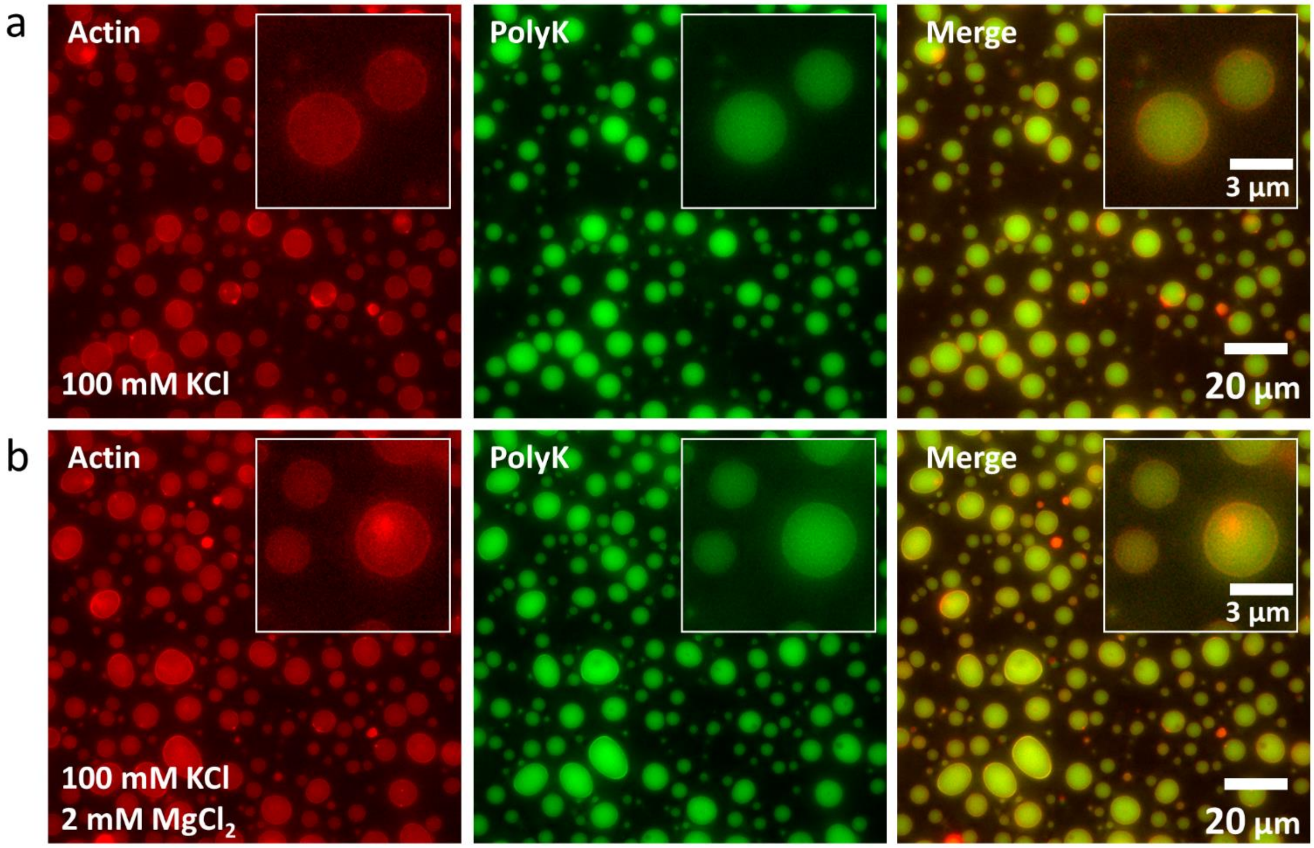

Supplementary Figure 2. Presence of KCl in the initial reaction mixture leads to homogenous partitioning of actin in the coacervates and further inhibits actinosome formation. (**a**) Coacervate droplets composed of polyK/NTP (*R* = 0.7) made in presence of KCl sequester the actin within the coacervates, as opposed to its localization at the interface in absence of KCl (Fig. 1). (**b**) Triggering actin polymerization by adding $MgCl_2$ does not result in actinosome formation. Images acquired in epifluorescence microscopy.

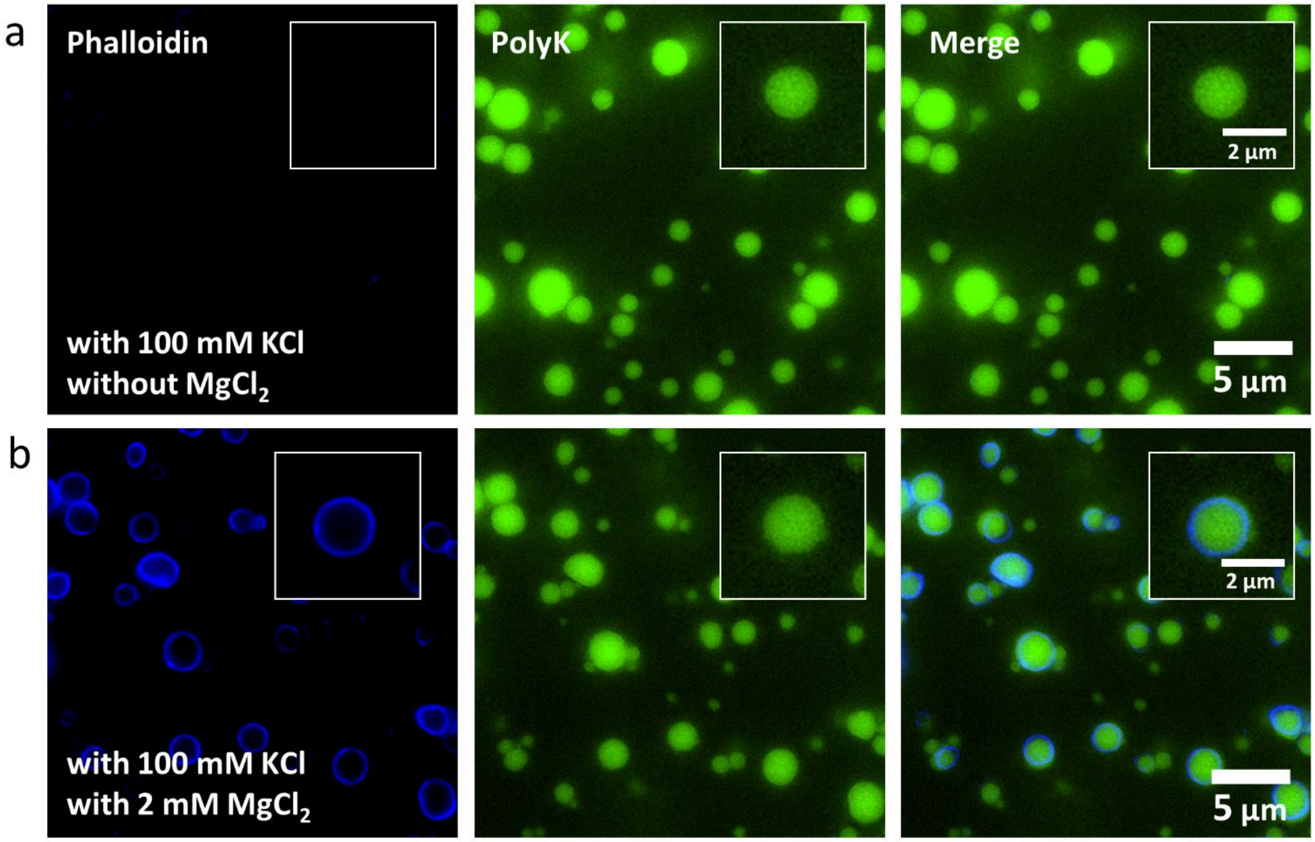

Supplementary Figure 3. Phalloidin staining of actin-coated condensates reveals actin polymerization on the coacervate surface in presence of Mg^{2+}. (**a**) Actin-coated coacervate droplets composed of polyK/NTP (*R* = 0.7) made in presence of KCl but without Mg^{2+} ions show absence of actin filaments. (**b**) In presence of both KCl and Mg^{2+} ions phalloidin stains the actin filaments polymerized on the surface of coacervates. Image contrast settings are the same for individual channels. Images acquired in epifluorescence microscopy.

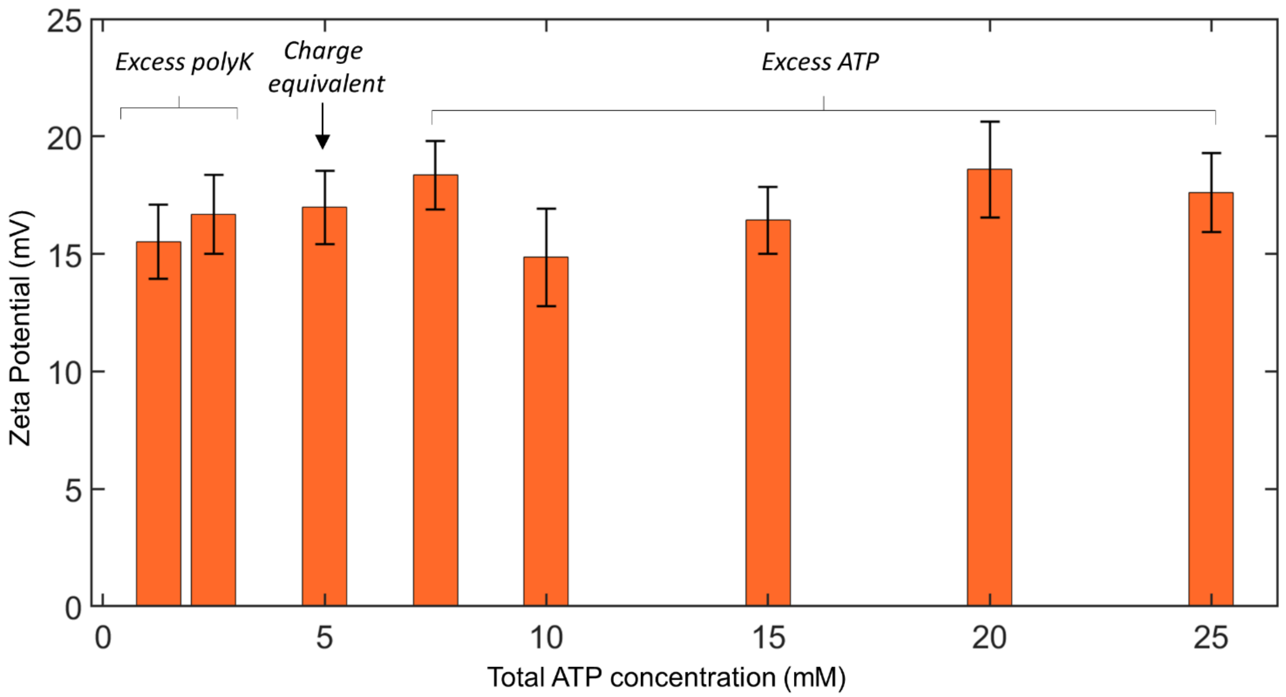

Supplementary Figure 4. Surface charge of polyK/ATP coacervates stays constant over different ATP: polyK ratios. Zeta potential of the polyK/ATP coacervate droplets was measured over a concentration range of 1.25 – 25 mM, with polyK concentration kept constant at 5 mg/mL. The measurements covered three regimes: excess polyK (positively charged polymer), charge equivalent state (net neutral), and excess ATP (negatively charged multivalent molecule). The values stayed relatively constant (16.9 ± 1.7 mV) over the entire ATP concentration range, suggesting a net positive surface charge on the coacervate droplets irrespective of limiting or excess ATP concentrations (*n* = 3 experimental repeats, with each measurement being an average of 5 individual runs; error bars indicate standard deviations).

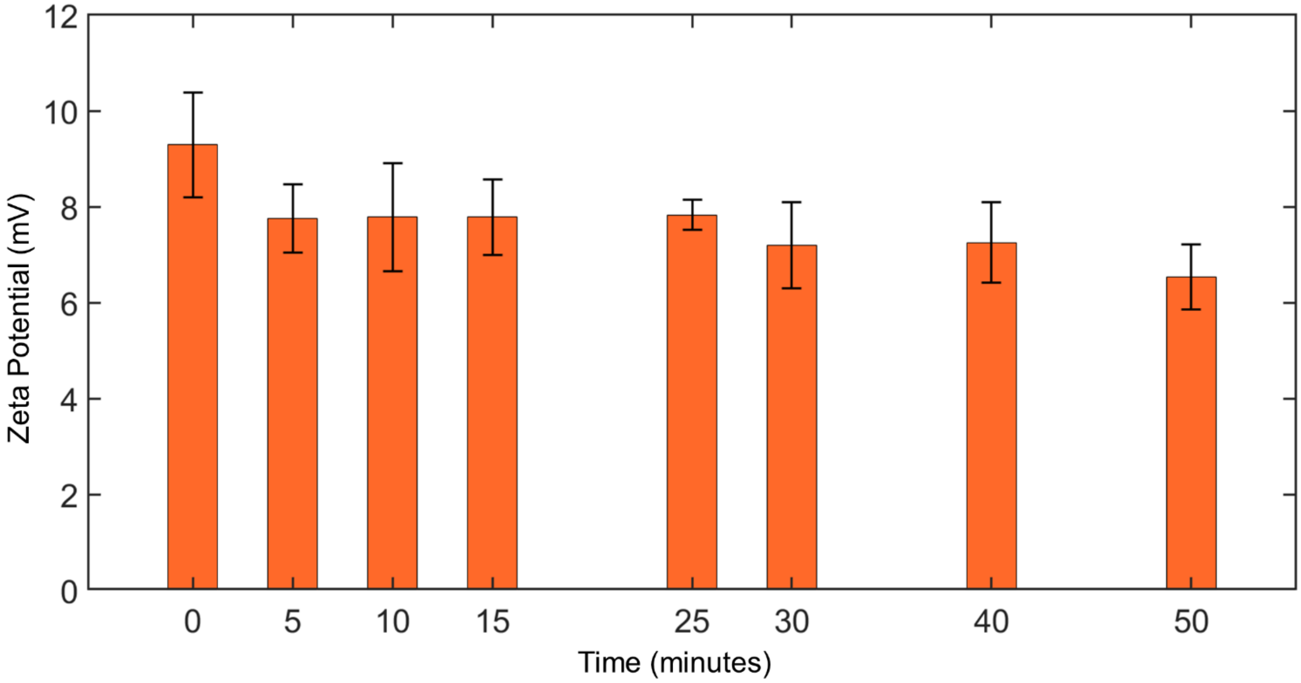

Supplementary Figure 5. Surface charge of polyK/ATP coacervates in presence of actin monomers over time. Zeta potential of polyK/ATP coacervate droplets was measured at different time points after the addition of actin. PolyK, ATP, and actin concentrations were respectively kept at 5 mg/mL, 5.4 mM, and 3 µM respectively; polymerization conditions were used. Immediately after the addition of actin, the surface charge was clearly lowered (< 10 mV) compared to when actin was absent (16.9 ± 1.7 mV; Supplementary Figure 3). This indicates efficient accumulation of actin at the surface. The surface charge was further decreased to 7.8 ± 0.7 mV after 5 minutes and then remained fairly constant over the entire time duration. (n = 3 experimental repeats, with each measurement being an average of 5 individual runs; error bars indicate standard deviations).

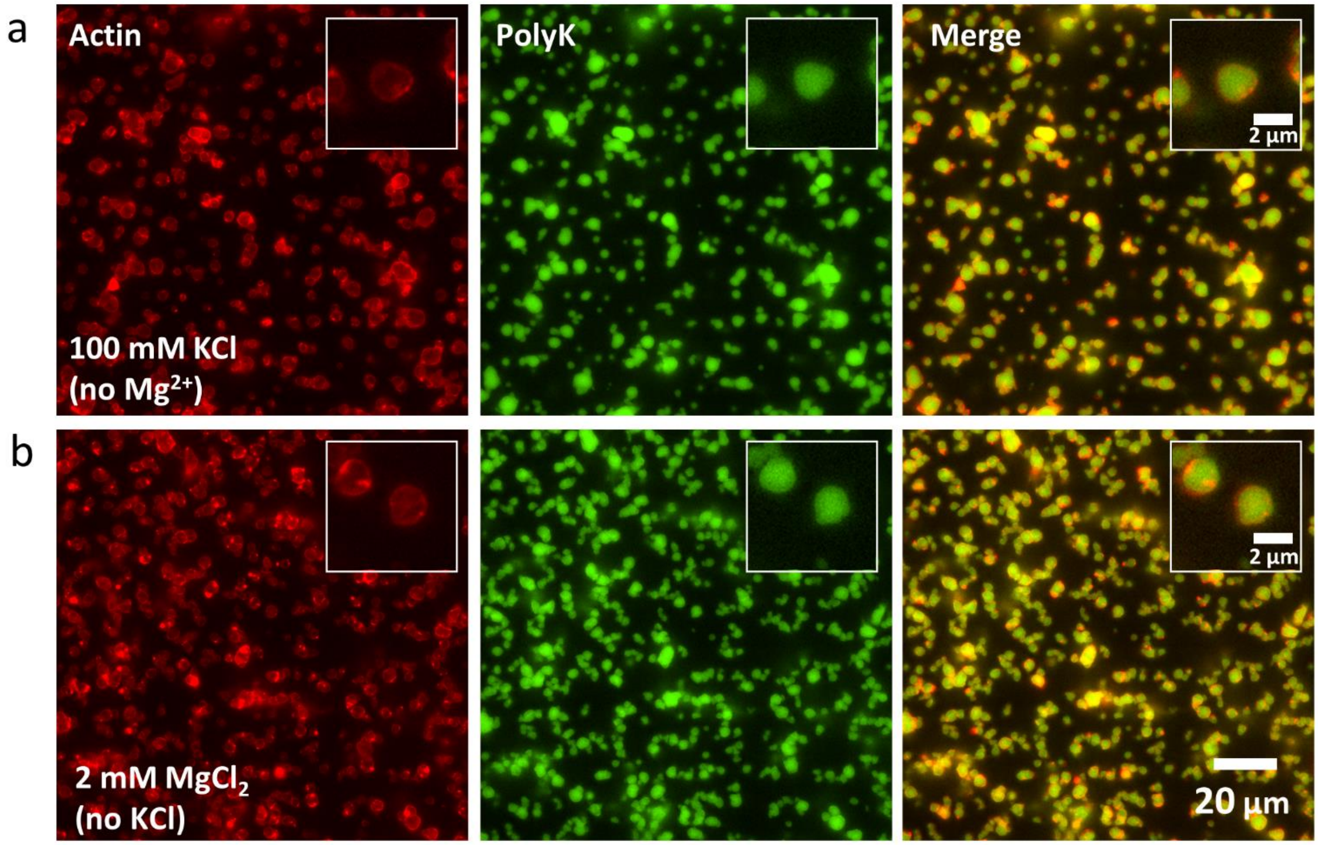

Supplementary Figure 6. Only a hyperosmotic shock (without actin polymerization) or only actin polymerization (without hyperosmotic shock) do not form actinosomes. (**a**) Coacervate droplets composed of polyK/NTP (R = 0.7) made in presence actin monomers lead to actin-coated condensates but resist coacervate dissolution and actinosome formation despite KCl-induced hyperosmotic shock. (**b**) Coacervate droplets composed of polyK/NTP (R = 0.7) form actin-coated condensates but do not lead to actinosomes after the addition of Mg^{2+} but without any hyperosmotic shock, despite triggering actin polymerization. Images acquired in epifluorescence microscopy.

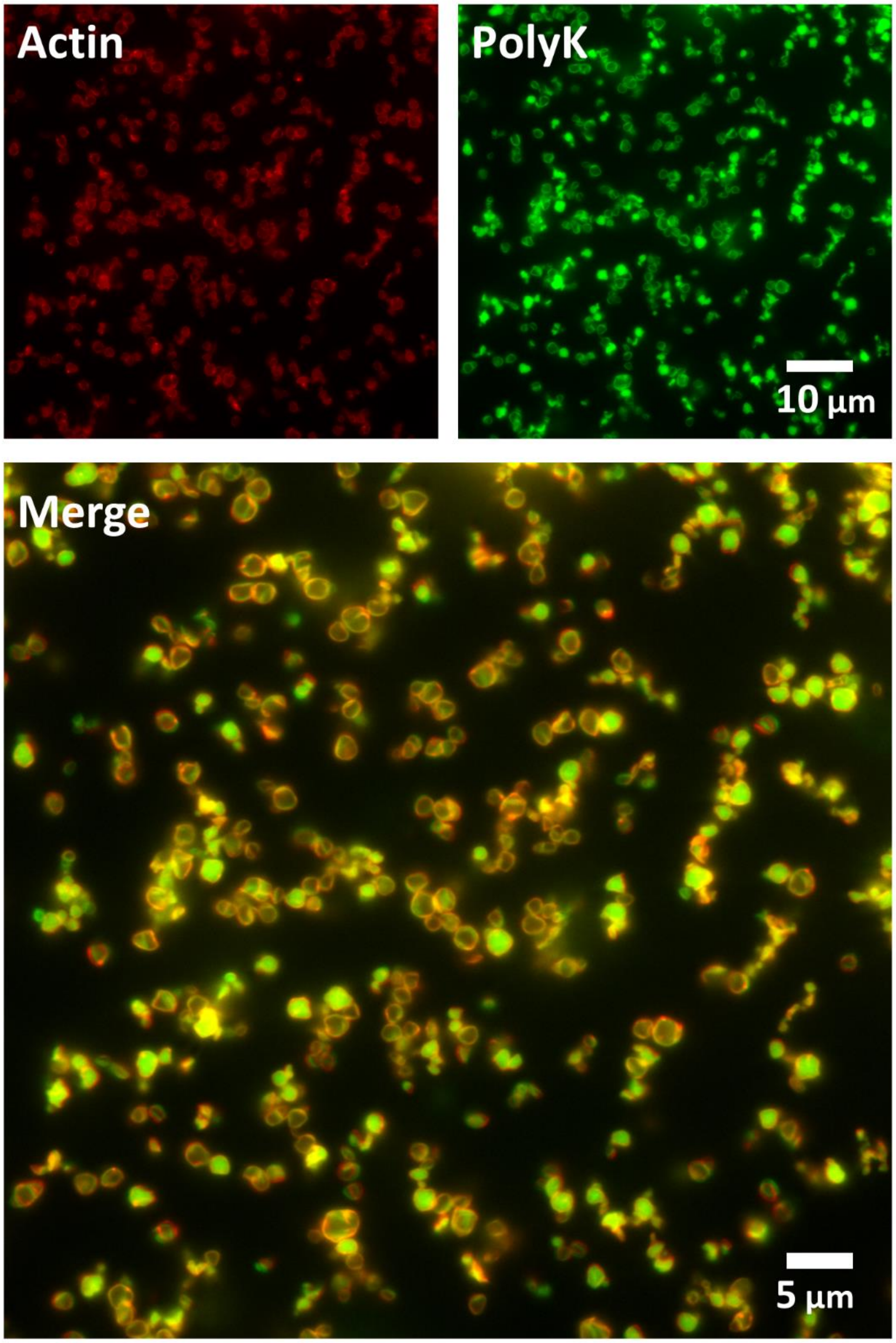

Supplementary Figure 7. Efficient actinosome formation. Full field-of-view of actinosomes synthesized using actin-coated polylysine/NTP condensates (R = 0.7). Images acquired in epifluorescence microscopy.

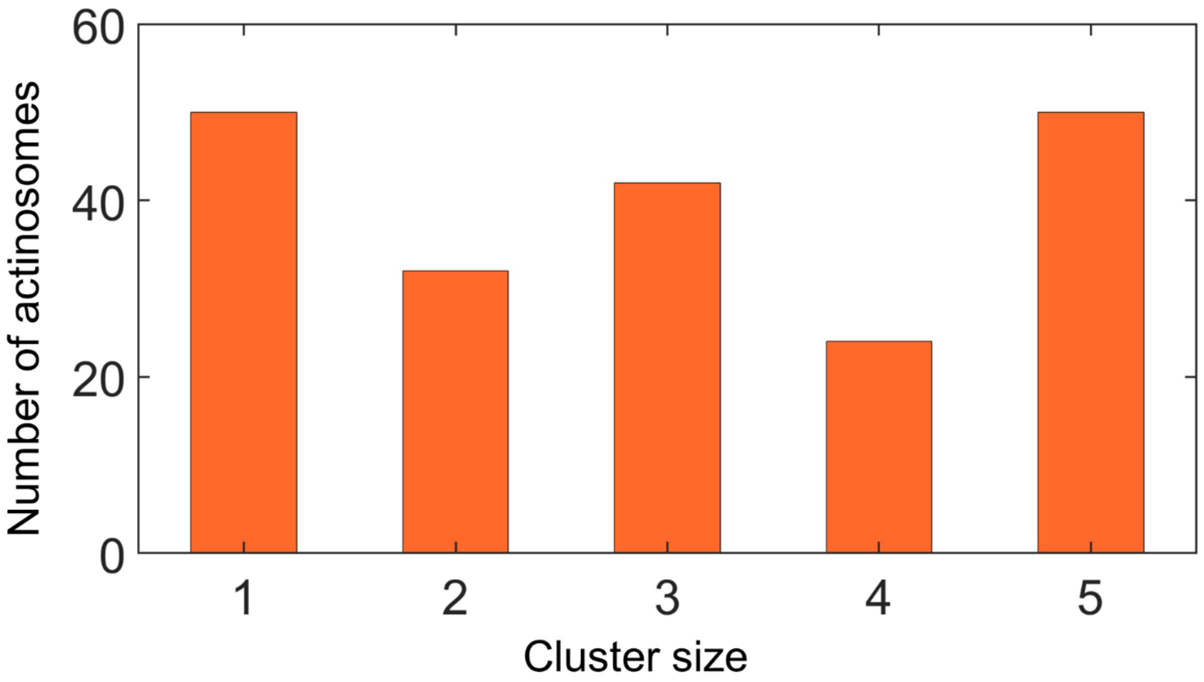

Supplementary Figure 8. Actinosomes show tendency to cluster together. Quantitative analysis of actinosomes (*n* = 198) show the tendency of actinosomes to form clusters of 2–5 units. Approximately 75% of actinosomes are in clustered state.

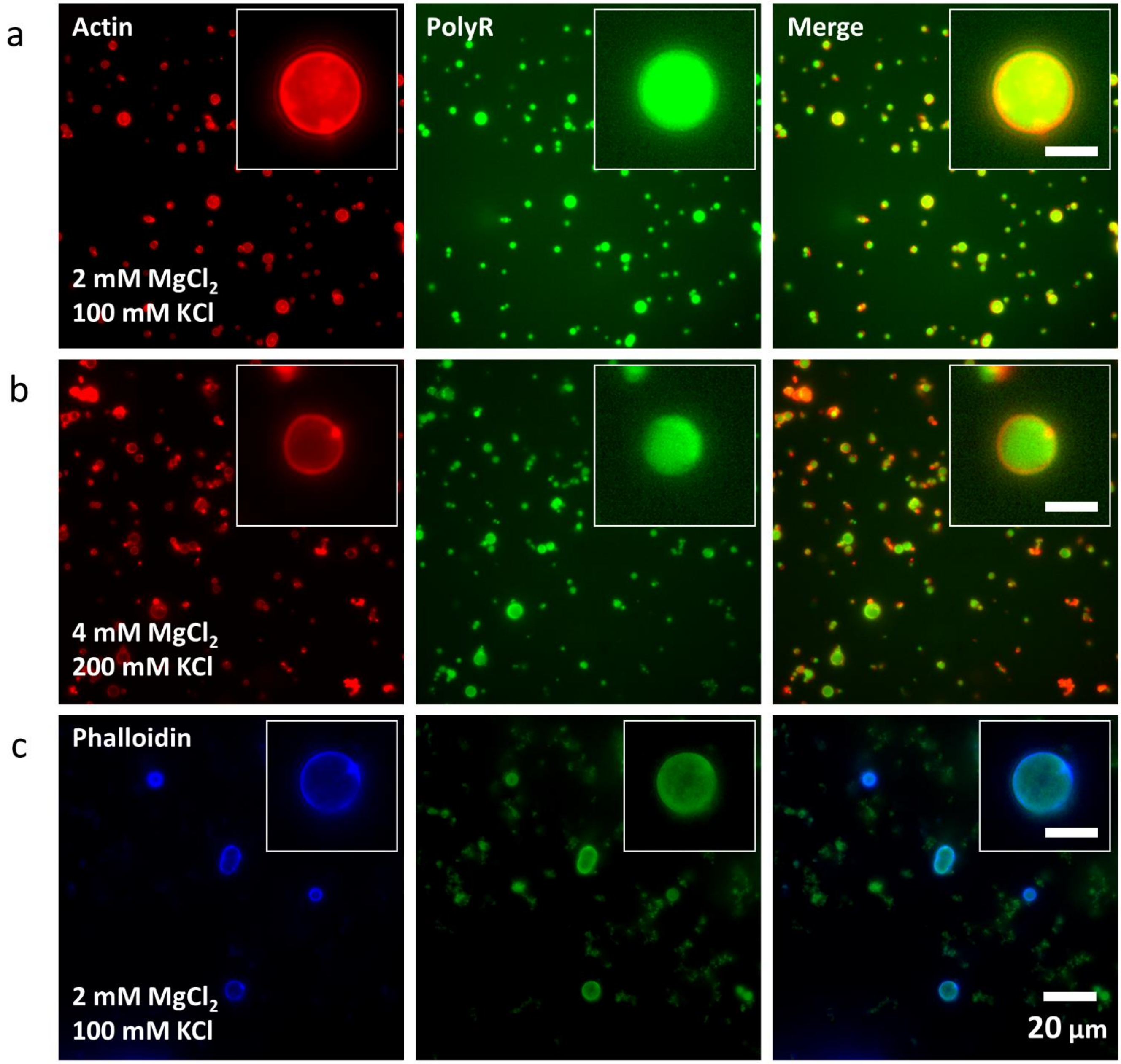

Supplementary Figure 9. PolyR/NTP coacervates do not form actinosomes. Coacervate droplets composed of polyR/NTP (R = 0.7) do not form actinosomes at similar buffer conditions (**a**) and even after doubling the concentrations of Mg^{2+} and KCl (**b**). Phalloidin staining confirms actin filament formation on the surface of the coacervates (**c**). Images acquired in epifluorescence microscopy.

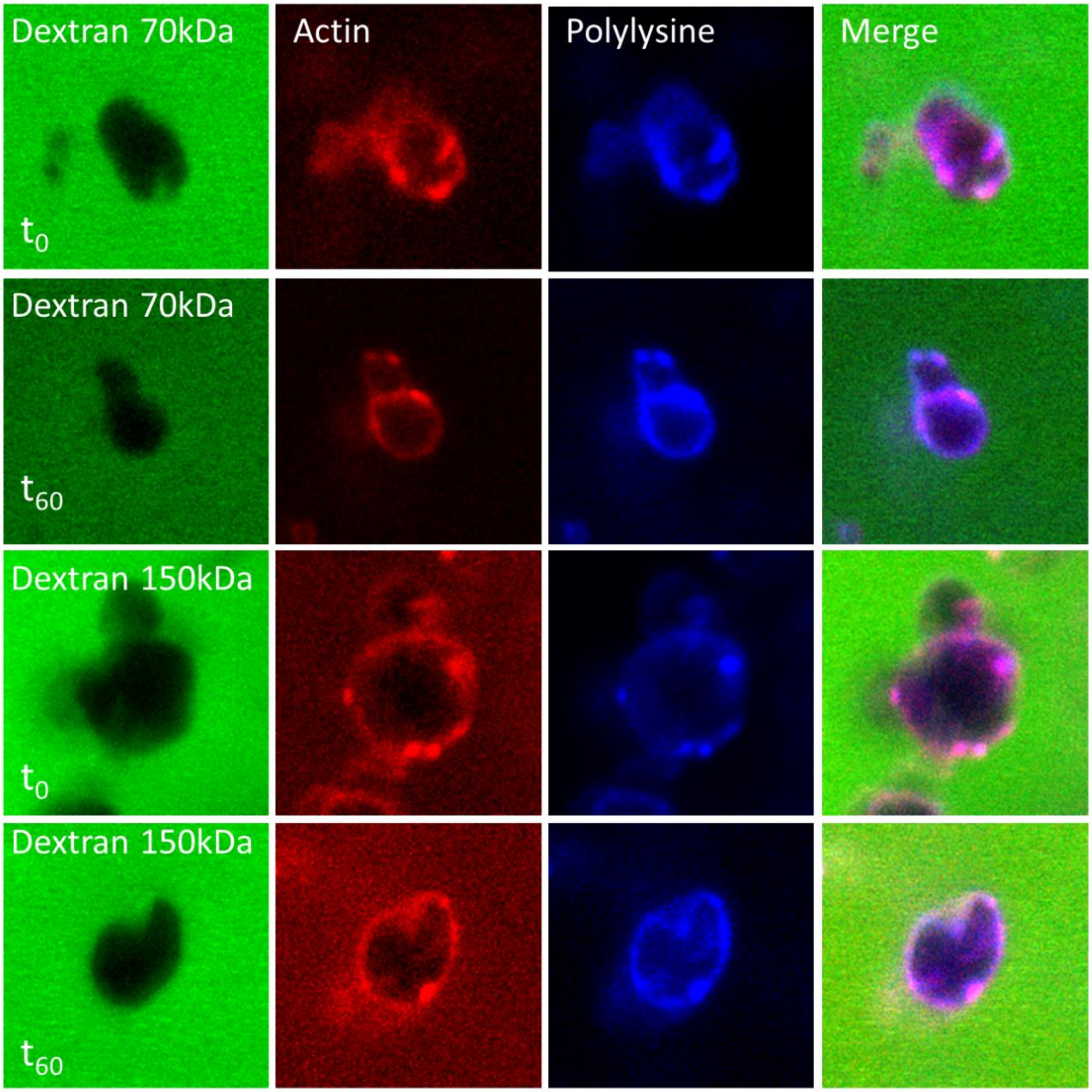

Supplementary Figure 10. Actinosomes are impermeable to high molecular weight dextran. Confocal images of pre-made actinosomes incubated with high molecular dextran, 70 kDa and 150 kDa corresponding to the diameter of gyration (Dg) of 5.45 and 7.02 nm respectively, showing the exclusion of dextran molecules within the actinosome interior. Two time slots are shown: right after the incubation (t_0) and after 60 minutes (t_{60}). Images acquired in confocal microscopy.

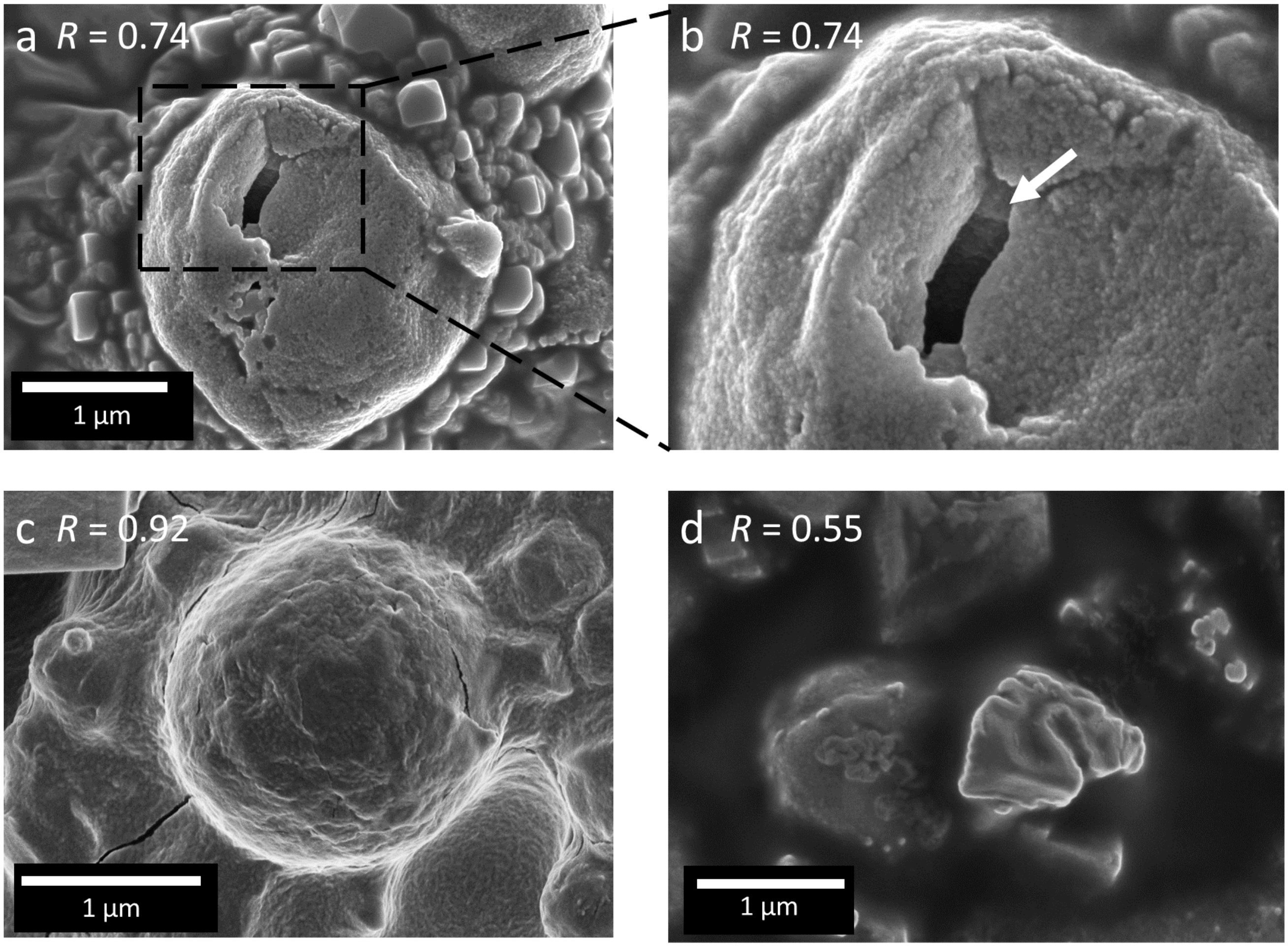

Supplementary Figure 11. Scanning electron microscopy of actinosome at varying NTP ratio. (a) Actinosome (R= 0.74) revealed a broken surface during the process of vacuum drying. (b) Zoom in showing a hollowness in actinosome interior. (c) Actin-coated condensates (R =0.92) showing a spherical morphology and a smooth surface without any pores or structuration. (d) Crumpled actin-polylysine structure (R= 0.55) because of high ATP concentration present in the condensates. The obtained structure is comparatively smaller and shows several folded surfaces.

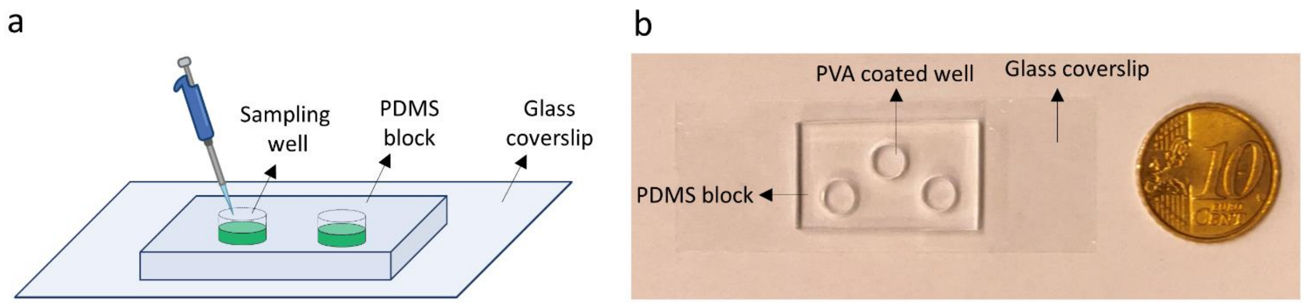

Supplementary Figure 12. Experimental setup for the visualization of actinosomes. PDMS block with wells (5 mm diameter) was bonded on a glass coverslip and the resulting well was coated with polyvinyl alcohol (PVA). (**a**) A schematic; (**b**) An actual device with three wells.

T7 Promotor

CCACTGCTTACTGGCTTATCGAAATTAATACGACTCACTATAGGGAGACCCAAGCTGGCTAGCGTTTAAACTTAAGCTTGGTACCGAGCTCGGATCCACTAGTCCAGTGTGGTGGAATTCA

GFP encoding

ACATGGTGAGCAAGGGCGAGGAGCTGTTCACCGGGGTGGTGCCCATCCTGGTCGAGCTGGACGGCGACGTAAACGGCCACAAGTTCAGCGTGTCCGGCGAGGGCGAGGGCGATGCC

ACCTACGGCAAGCTGACCCTGAAGTTCATCTGCACCACCGGCAAGCTGCCCGTGCCCTGGCCCACCCTCGTGACCACCCTGACCTACGGCGTGCAGTGCTTCAGCCGCTACCCCGACCAC

ATGAAGCAGCACGACTTCTTCAAGTCCGCCATGCCCGAAGGCTACGTCCAGGAGCGCACCATCTTCTTCAAGGACGACGGCAACTACAAGACCCGCGCCGAGGTGAAGTTCGAGGGCG

ACACCCTGGTGAACCGCATCGAGCTGAAGGGCATCGACTTCAAGGAGGACGGCAACATCCTGGGGCACAAGCTGGAGTACAACTACAACAGCCACAACGTCTATATCATGGCCGACAA

GCAGAAGAACGGCATCAAGGTGAACTTCAAGATCCGCCACAACATCGAGGACGGCAGCGTGCAGCTCGCCGACCACTACCAGCAGAACACCCCCATCGGCGACGGCCCCGTGCTGCTG

CCCGACAACCACTACCTGAGCACCCAGTCCGCCCTGAGCAAAGACCCCAACGAGAAGCGCGATCACATGGTCCTGCTGGAGTTCGTGACCGCCGCCGGGATCACTCTCGGCATGGACG

AGCTGTACAAGTAAGTCTAGAGGGCCCGTTTAAACCCGCTGATCAGCCTCGACTGTGCCTTCTAGTTGCCAGCCATCTGTTGTTTGCCCCTCCCCCGTGCCTTCCTTGACCCTGGAAGGTG

CCACTCCCACTGTCCTTTCCTAATAAAATGAGGAAATTGCATCGCATTGTCTGAGTAGGTGTCATTCT

Supplementary Figure 13. DNA template used for GFP production. Template DNA encoding green fluorescent protein (highlighted in green) under the bacterial T7 promotor (highlighted cyan) that was used for cell-free *in vitro* synthesis of mRNA.

Supplementary Movie legends

Supplementary Movie 1

Time-lapse showing the crumpling of polylysine/ATP condensate (green; R = 0) caused by a combination of hyperosmotic shock and ATP depletion via actin polymerization on the surface (red).

Supplementary Movie 2

Time-lapse showing the process of polylysine (green) getting expelled from actin-coated polylysine/ATP/GTP condensate (R = 0.7) due to hyperosmotic shock (100 mM KCl) to form an actinosome.

Supplementary Movie 3

Z-stack of an actinosome (R = 0.7). The two main components of actinosome, polylysine and actin are colored in green and red respectively.

Supplementary Movie 4

Time lapse showing polylysine/GTP condensate (green; R = 1) coated with actin (red) remaining stable over time.

Supplementary Movie 5.

Time-lapse showing the expression of GFP protein (green) by encapsulating GFP-encoding mRNA and *in vitro* translation machinery inside actinosomes (red; R = 0.7). GFP expression was imaged in real time by incubating the sample at 29°C using a heating stage mounted on the microscope.
